# Transcription Start Site Associated RNAs (TSSaRNAs) Are Ubiquitous in All Domains of Life

**DOI:** 10.1371/journal.pone.0107680

**Published:** 2014-09-19

**Authors:** Livia S. Zaramela, Ricardo Z. N. Vêncio, Felipe ten-Caten, Nitin S. Baliga, Tie Koide

**Affiliations:** 1 Department Biochemistry and Immunology, Ribeirão Preto Medical School, University of São Paulo, Ribeirão Preto, Brazil; 2 Department of Computing and Mathematics, Faculdade de Filosofia Ciências e Letras de Ribeirão Preto, University of São Paulo, Ribeirão Preto, Brazil; 3 Institute for Systems Biology, Seattle, Washington, United States of America; Niels Bohr Institute, Denmark

## Abstract

A plethora of non-coding RNAs has been discovered using high-resolution transcriptomics tools, indicating that transcriptional and post-transcriptional regulation is much more complex than previously appreciated. Small RNAs associated with transcription start sites of annotated coding regions (TSSaRNAs) are pervasive in both eukaryotes and bacteria. Here, we provide evidence for existence of TSSaRNAs in several archaeal transcriptomes including: *Halobacterium salinarum*, *Pyrococcus furiosus*, *Methanococcus maripaludis*, and *Sulfolobus solfataricus*. We validated TSSaRNAs from the model archaeon *Halobacterium salinarum* NRC-1 by deep sequencing two independent small-RNA enriched (RNA-seq) and a primary-transcript enriched (dRNA-seq) strand-specific libraries. We identified 652 transcripts, of which 179 were shown to be primary transcripts (∼7% of the annotated genome). Distinct growth-associated expression patterns between TSSaRNAs and their cognate genes were observed, indicating a possible role in environmental responses that may result from RNA polymerase with varying pausing rhythms. This work shows that TSSaRNAs are ubiquitous across all domains of life.

## Introduction

Molecular mechanisms that are conserved throughout evolution, or arise independently to perform similar tasks are of major interest to biology [1]. Evolutionary conservation and convergence are strong indicators of important biological functions. Understanding commonalities and differences across organisms from all three domains of life have therefore served as powerful means to discover and characterize important molecular mechanisms.

The roles of non-coding RNA (ncRNA) molecules have proven to be especially elusive. Only recently, high-throughput technologies have revealed that ncRNAs have important functions across diverse biological systems and processes [2], [3]. Among the newly discovered ncRNAs is an intriguing class of transcription start site associated RNAs (TSSaRNAs) that have thus far been observed in eukaryotes and bacteria [4]–[7].

Based on their location, TSSaRNAs have been speculated to play a role in transcription initiation [5], [6], [8]; and based on their tissue-specific regulation they have also been putatively implicated in epigenetic regulation [5], [9]. TSSaRNAs have also been reported in bacteria where it is suggested that they could be part of a regulatory mechanism that prevents transcription initiation until a functional RNA polymerase complex has assembled [4]. In both eukaryotes and bacteria, the production of these transcripts seems to be associated with stalled RNA polymerase [4]–[6]. The RNA polymerase pausing model is the most accepted TSSaRNA biogenesis hypothesis and its functional implications is still under investigation [10], [11].

Regardless, TSSaRNA ubiquity across eukaryotes and bacteria suggests that TSSaRNAs are ancient and must have been present in LUCA. Discovery of TSSaRNAs in archaea would lend credibility to this hypothesis and provide clues into why they are evolutionarily conserved across all organisms.

## Results and Discussion

### Discovery of TSSaRNAs in the third domain of life

**Figure 1:**
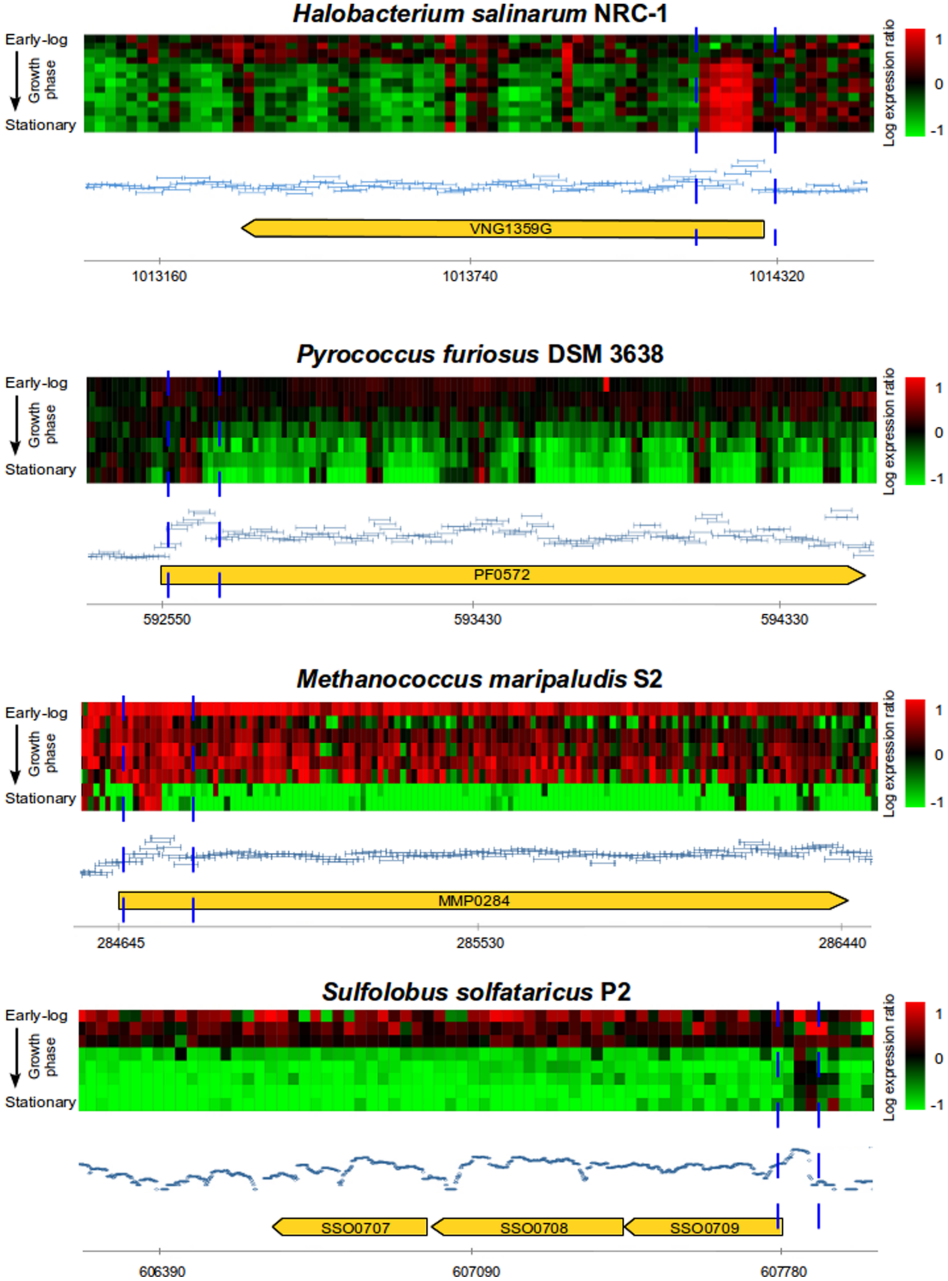
Data-mining on archaeal gene expression public datasets. Each panel shows an example of TSSaRNA presence in different archaeal transcriptomes. Yellow arrow represents the cognate gene, blue dashed lines represent TSSaRNA estimated regions and horizontal axis represent organism's genome coordinates. Heatmaps represent gene expression profiles over growth curves and are color-coded according to log_10_ expression ratios between each time point relative to reference condition. Light blue horizontal bars represent tiling array probe intensities for reference conditions for *H. salinarum*, *P. furiosus* and *M. maripaludis*. Dark blue points (*S. solfataricus* only) represent RNA-seq reads coverage data. Figure S1 show examples for additional archaea.

In the present work, we investigated whether TSSaRNAs do indeed exist in archaea and, thus, ubiquitous across all three domains of life. By mining publicly available data, we gathered evidence for TSSaRNAs in 10 archaeal transcriptomes (*H. salinarum*, *M. maripaludis*, *S. solfataricus*, *P. furiosus*, *N. equitans*, *M. kandleri*, *H. volcanii*, *M. psycrophilus*, *M. mazei* and *P. abyssi* [12]–[21], see supplemental material), including compendia of gene expression profiles over growth curves for 4 organisms: *H. salinarum* [14], *M. maripaludis* [12], *S. solfataricus* [12], [13] and *P. furiosus* [12] (**Figure 1**). We mined publicly available gene expression datasets from GEO [22] (http://www.ncbi.nlm.nih.gov/geo/), SRA [23] (http://www.ncbi.nlm.nih.gov/sra) and UCSC Archaeal Genome Browser [24] (http://archaea.ucsc.edu/). Datasets not available in public databases were obtained directly from publications.

Expression of a putative TSSaRNA, measured either by hybridization intensities or by read coverage, had a distinct signature characterized by a sharp rise in signal that plateaus over a small distance and then decays precipitously. This signature was conserved across most transcriptomes that were analyzed, and across all sequencing (Illumina, SOLiD and Roche 454) and microarray (NimbleGen and Agilent) platforms, and all library construction protocols (strand-specific and non-strand specific) [12]–[21] (Figure S1). Aiming TSSaRNAs discovery in all archaeal organisms, all datasets were manually inspected.

### TSSaRNAs in *H. salinarum* NRC-1

The consistency of TSSaRNAs discovery across all platforms and organisms justified further experimentation for independent validation. *H. salinarum* is a model organism for halophilic archaea and has been extensively studied in the last decade. It became a prime model to study aspects of gene expression regulation, especially due to the establishment of predictive quantitative models with high accuracy [25].

In order to precisely map TSSaRNAs in *H. salinarum* NRC-1, we performed a strand-specific RNA-seq experiment using non-fragmented small RNAs in the 20–230 bp range. Two biological replicates were extracted from cultures under standard growth conditions [26]. From these replicates, 3.4 million reads were aligned to *H. salinarum* NRC-1 genome.

**Figure 2:**
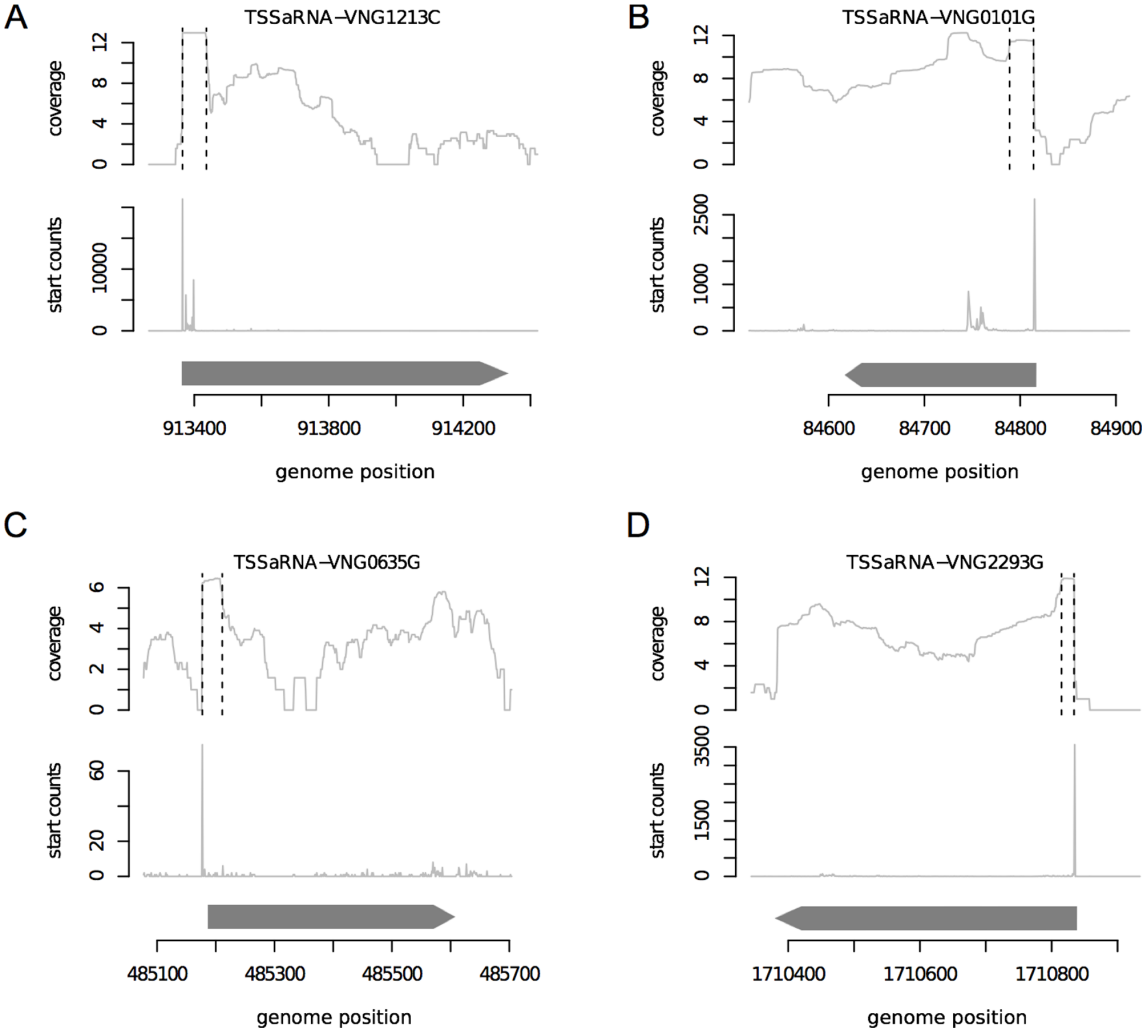
Transcriptome mapping using small RNA sequencing. Strand-specific RNA-seq experiment was performed using non-fragmented small RNAs (20–230 pb). Panels A–D show examples of TSSaRNAs and their cognate genes. (A) VNG1213C, forward strand (B) VNG0101G, reverse strand (C) VNG0635G, forward strand. (D) VNG2293G, reverse strand. For each panel, the genome position and CDSs location and orientation (grey arrows) are indicated at the horizontal axis. The uppermost graphic show log_2_ transformed amount of reads covering a given genomic coordinate. Vertical dashed lines represents the TSSaRNA region. The intermediary graphic represents the amount of aligned reads whose start position maps to a given genomic coordinate, the “start profile”.

The reads from TSSaRNAs create a surplus in coverage values when taken together with reads from the cognate gene (**Figure 2** **, Figure S2**). A given genomic location can have two sets of aligned reads starting exactly there: (i) reads from transcripts greater than 151 nt but truncated at any length, up to the maximum sequencing length limit (151 bp) and (ii) identical full-length reads from transcripts smaller than 151 nt. Although both sets map to the same initial position, the former show repeatedly the same start and end genomic coordinates. We used relative enrichment of the aligned start position as a feature to automatically detect TSSaRNAs (peaks in “start counts” profiles in **Figure 2**). Using this approach, we discovered 652 TSSaRNAs that were evenly distributed on both strands, and associated with 25% of all annotated protein coding genes.

To distinguish between processed and primary transcripts, we performed a dRNA-seq experiment [27]. Since primary transcripts have their 5′ ends intact, a TEX (Terminator 5′-Phosphate-Dependent Exonuclease) enzyme treatment would enrich a sample for them. Comparing sequenced reads from treated (TEX+) and control (TEX-) libraries it is possible to identify primary TSSaRNAs. Using this approach, we refined our observations and defined 179 primary TSSaRNAs that were evenly distributed on both strands, and associated with 7% of all annotated protein coding genes (**Table S1**). It is important to note that dRNA-seq experiments are prone to false negatives [28]–[29], thus, it is possible that more than 179 TSSaRNAs do exist. To turn the association of TSSaRNAs to transcription start sites (TSS) robust, we choose to further investigate only those small RNAs strictly correlated to primary TSS positions validated by dRNA-seq data.

The TSSaRNA sizes in *H. salinarum* ranged from 16 nt to 146 nt with a median size of 27 nt (**Figure S3A**). The distribution and median size of TSSaRNAs was consistent across many organisms: murine (range: 20 nt to 90 nt, median 20 nt) [6]; human, chicken and fruit fly (range in all three: 13 nt to 28 nt, median 18 nt) [5]. By contrast, the distribution of TSSaRNA sizes in some bacterial organisms was much narrower, e.g., *E. coli* (range: 33 nt to 40 nt) and *M. pneumoniae* (range: 35 to 55 nt, few TSSaRNAs up to ∼100 nt) [4]. The proximal locations of TSSaRNAs to translation initiation sites of cognate genes (**Figure S3B**) are consistent with previous observations that most transcripts in *H. salinarum* are leaderless [14]. As for bacteria and eukaryote, the distribution of TSSaRNAs location shows that there are some TSS internal to annotated CDSs, which may point to structural annotation imprecision or alternative transcripts.

### Transcriptome data indicates multiple and time-varying RNA polymerase pausing sites

The current understanding is that the production of TSSaRNA transcripts is associated with stalled RNA polymerase during cognate gene transcription in eukaryote and bacteria [4]–[6]. This polymerase pausing hypothesis is becoming the prime biogenesis model for TSSaRNA and is bringing key insights into gene expression regulation [10], [11], eclipsing alternative hypothesis such as degradative 3′ end processing or non-degradative (cleavage) gene processing.

In archaea, the absence of a set of RNA-seq reads starting just before TSSaRNA reads' ends (**Figure S4**) argue against the cleavage biogenesis hypothesis. Moreover, the observation that TSSaRNA compositional/thermodynamical properties are no different from similar regions in non-cognate gene sequences (**Figure S5**) argue against the degradative biogenesis hypothesis, following the same rationale put forward by Yus *et al.* [4]. Unsurprisingly, given that the molecular mechanisms involved in RNA polymerase pausing are complex [27] and often involve gene specific structures [28], there were no clear pausing site signatures in the vicinity of all 179 primary TSSaRNA 3′ ends, or even considering all 652 putative TSSaRNAs. Altogether, we have no evidence to believe that only archaea would present a different biogenesis process other than RNA polymerase pausing. To explore this hypothesis properties, we created a simple computational model for RNA polymerase pausing biogenesis scenario (**File S1, File S2**). This model explores only two parameters for RNA polymerase: elapsed time paused at any given genomic location and time between successive transcription initiation events (**Figure S6**).

**Figure 3:**
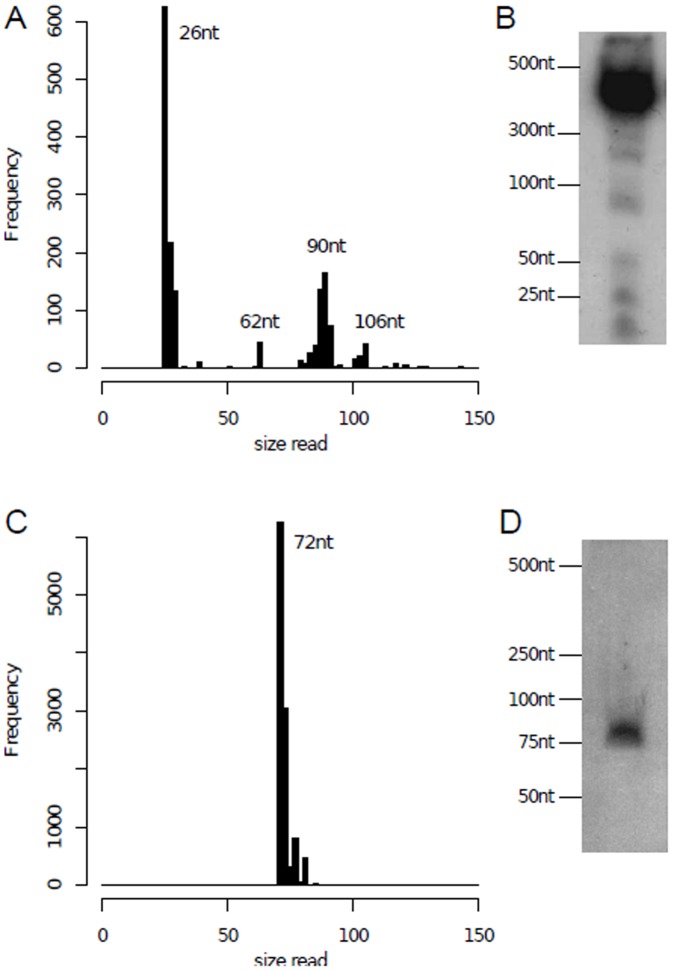
TSSaRNAs reads size distribution and northern blot validation. Panel A shows the size histogram of all reads that aligned their 5′ ends at VNG0101G's TSS. It is possible to verify an enrichment of sizes of 26 nt, 62 nt, 90 nt and 106 nt, which corresponds to the bands observed in the northern blot experiment. Panel B shows northern blot analysis for TSSaRNA associated with VNG0101G. Panel C shows the size histogram of all reads that aligned their 5′ ends at VNG1213C's TSS. Panel D shows northern blot analysis for TSSaRNA associated with VNG1213C.

Using multiple pausing sites along a gene with different retention times, the model explains a recurrent RNA-seq experimental observation in our datasets: an ensemble of full-length reads aligned at the same starting position, but with different sizes. We validated this model's implication by performing classical northern-blot experiments for two highly expressed genes: one showing signs of multiple pausing sites (VNG0101G) and one derived from a single pausing site (VNG1213G). VNG0101G encodes a conserved cold shock protein and was selected for further validation since the signal associated with its TSSaRNA was top ranked in tiling array experiments [14]. Notwithstanding the low sensitivity of detecting low abundance RNAs with northern blot [5], the 26 nt TSSaRNA was observed as a distinct band along with its cognate gene transcript (**Figure 3B**). Along with the northern-blot band directly corresponding to the most frequent reads aligned at VNG0101G's TSS position (**Figure 3A**), it is possible to see other less stronger bands, which sizes also correspond to less abundant RNA-seq reads. The computational model can easily recapitulate these observations by using multiple retention positions and times (**File S1**). If, on the other hand, only one genomic position stalls a RNA polymerase, then only one type of small molecule associated with the TSS would be created. This case is also observed experimentally for VNG1213C gene, a probable exonuclease: RNA-seq data shows a population of reads concentrated around 72 nt, which maps directly with the single band found in the northern-blot experiment (**Figure 3CD**). Therefore, our transcriptome data indicates that it is possible to find multiple RNA polymerase pausing sites along a gene sequence.

**Figure 4:**
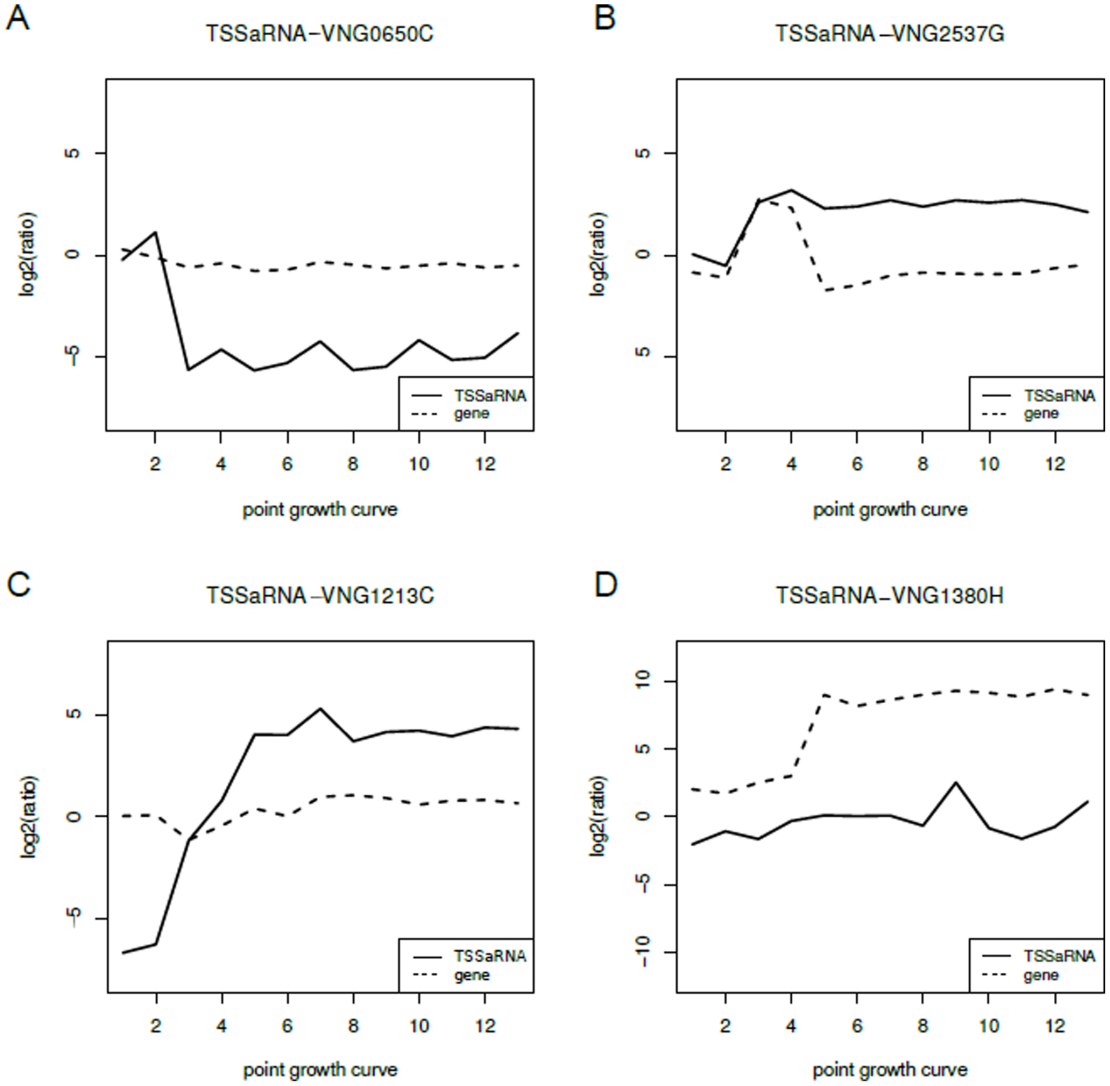
TSSaRNAs differentially expressed. Panels A, B, C and D are expression profiles of TSSaRNAs differentially expressed over a typical growth-curve relative to the control growth condition. Horizontal axis represent 13 growth curve points from different phases in a standard laboratory batch culture. Solid lines are TSSaRNA expression profiles and dashed lines their cognate gene expression profile.

Remarkably, it was clear from gene expression profiles that dynamical behavior of a TSSaRNA may be distinct from that of its cognate gene. In some cases, the cognate gene level does not change, but expression of the TSSaRNA has distinct dynamics, with up to 16 fold up-regulation or down-regulation to different degrees (**Figure 4AC**). We also observed instances when both TSSaRNA and cognate gene were differentially regulated, albeit with different patterns (**Figure 4BD**). Imposing stringent criteria, we identified at least 10 TSSaRNA differentially expressed relative to their cognate genes (**Table S2, Figure S7**). Such differential expression patterns would not be expected if transcription of a TSSaRNA and the full-length transcript of its cognate gene were not regulated by environmental signals, nor could it arise as an experimental artifact of tiling array hybridization and processing. Using pausing sites that can vary their retention time along the growth curve, the RNA polymerase pausing model explains our experimental observation that TSSaRNA can have distinct dynamical behavior relative to their cognate gene. Although counterintuitive, it is possible to generate dynamical profiles such as the ones where TSSaRNA levels remains constant and its cognate gene varies and *vice versa*, only exploring the two parameters of the model: elapsed time spent paused and time between successive transcripts initiation events (**Figure S8, File S2**).

Therefore, our transcriptome analysis indicates that there is probably RNA polymerase pausing rhythm regulation in response to environmental perturbations. Future experimental work would reveal how this rhythm may be tuned and what are the implication of this regulation.

## Conclusions

In this study we demonstrated that TSSaRNAs are also present in archaea. Our findings complement previous discoveries of these ncRNAs in eukaryotes and bacteria, to show that TSSaRNAs are ubiquitous in all domains of life. Furthermore, the northern-blot banding patterns in our experiment were consistent with previous observations in eukaryotes and bacteria [6], [30], suggesting that TSSaRNAs may be accompanied by a population of transcripts. The prevalent TSSaRNA biogenesis hypotheses, the RNA polymerase pausing, would easily explain these patterns as well as our observation of TSSaRNA/cognate gene differential expression. Comparative transcriptome analysis among all domains of life will be critical for elucidating the precise roles played by TSSaRNAs, in order to explain why they are evolutionarily conserved.

## Materials and Methods

### Data-mining on archaeal gene expression datasets

To verify the presence of TSSaRNAs in archaea, we mined archaeal publicly available gene expression datasets from GEO [22], SRA [23] and UCSC Archaeal Genome Browser [24].

In this study we analyzed the transcriptome of 11 archaea: Halobacterium salinarum NRC-1, Pyrococcus furiosus DSM 3638, Methanococcus maripaludis S2, Sulfolobus solfataricus P2, Nanoarchaeum equitans Kin4-M, Methanopyrus kandleri AV19, Sulfolobus acidocaldarius MW001, Haloferax volcanii DS2, Methanolobus psycrophilus R15, Methanosarcina mazei Gö1 and Pyrococcus abyssi [12]–[21]. Only S. acidocaldarius data did not present sufficient coverage to clearly show at least one TSSaRNAs signature. Therefore, our observations were made for 10 organisms. Archaeal transcriptomes for which dynamical information was available were highlighted in this work: Halobacterium salinarum NRC-1 [14], Pyrococcus furiosus DSM 3638 [12], Methanococcus maripaludis S2 [12] and Sulfolobus solfataricus P2 [12], [13]. Original accession numbers for these datasets are: GSE13150, GSE18630, GSE38821, GSE26782, GSE44979, SRP028191, SRX188664. Datasets not available in public databases were obtained directly from publications. A brief description for each dataset used is provided in the **Table S3**.

The expression signal for putative TSSaRNAs locations is a distinct signature characterized by a sharp rise in signal that plateaus over a relatively small distance and then decays precipitously. Tiling array probe intensities and log ratio data for all growth curve time points were obtained from GEO and processed as described in [14]. Heatmaps for expression profiles over the growth curve were relative to a reference growth condition and visualized in Gaggle Genome Browser [31]. Raw RNA-seq datasets were processed by: i) trimming each library using FASTX-toolkit (http://hannonlab.cshl.edu/fastx_toolkit/) to remove adapters; ii) mapping against appropriate reference genomes using Bowtie [32]; and iii) visualizing non-normalized reads coverage as a proxy for gene expression using the integrative tool Gaggle Genome Browser [31].

### Cell cultivation and small RNA isolation

*H. salinarum* NRC-1 was grown in CM media, in a water bath incubator at 37°C with agitation of 125 r.p.m. Reference samples were cultured under standard growth conditions [26], at mid-log phase (OD600≈0.5). Small RNAs for RNA-seq libraries and Total RNAs for dRNA-seq libraries and northern blot experiments were isolated using the MirVana RNA extraction kit (Ambion).

### RNA-seq library preparation, sequencing and pre-processing

Two small RNA libraries (biological replicates) from *H. salinarum* NRC-1 were prepared for sequencing. Small RNAs at mid-log phase cultures were extracted. For each sample, 10 µg of small RNAs were treated with RNAse-free DnaseI (Fermentas) in a final volume of 30 µL. The reaction was incubated for 45 min at 37°C and the RNA was purified using phenol/chloroform purification. 1 µg of treated small RNA was ligated to RNA 3′ Adapter (RA3) using T4 RNA ligase 2 truncated (BioLabs) for 1 hour at 28°C, in the presence of RNase inhibitor. Once RA3 was ligated, we performed the RNA 5′ Adapter (RA5) ligation using T4 RNA ligase in the presence of 10 mM ATP. cDNA was synthesized using specific oligos for 5′ and 3′ adapters using SuperScript III Reverse Transcriptase, according to Illumina Truseq protocol. cDNA libraries were amplified and samples were separated in a Novex 6% PAGE gel. cDNAs from 20 bp up to 230 bp were isolated from the gel and subjected to quantification and quality analysis.

The resulting double stranded cDNA was sequenced on Illumina Miseq v2 platform. Biological replicates were sequenced in the same flow-cell using different indexes. Strand-specific sequencing was performed in MiSeq set to 151 cycles per manufacturer's instructions.

Reads were trimmed using FASTX-toolkit (http://hannonlab.cshl.edu/fastx_toolkit/links.html) to remove adapters. Processed reads were aligned against *H. salinarum* NRC-1 reference genome (chromosome: NC_002607, plasmid pNRC100: NC_001869.1 and plasmid pNRC200: NC_002608.1) using Bowtie [32] with default parameters (except by “m” parameter, since we discarded ambiguous alignment). Overall, 3,489,281 aligned reads from biological replicates combined were considered in subsequent analysis.

RNA-Seq data were submitted to NCBI's SRA website under the accession number **SRP035406**.

### TSSaRNA definition in *H. salinarum*

Since *H. salinarum* small RNA libraries were made without fragmentation, we can observe two sets of reads consistently aligned at the same position near the start codon of a gene: (i) reads marking the transcription start site (TSS) of the gene itself, truncated at diverse lengths up to 151 bp; and (ii) reads smaller than 151 bp consistently found with the same 3′ end, thus, being full-length reads (**Figure S9**). Type (ii) reads are generally generated by TSSaRNAs.

We used relative enrichment of reads' aligned start coordinates as a parameter to automatically detect TSSaRNAs. We looked for the most frequent start coordinate near the start codon of a CDS. The search was performed in a window starting 50 bp upstream of the translation start site and comprising at maximum 20% of CDS length. To make sure that the TSS is reliable, reads must sum up more than 20 counts. This procedure can detect TSSs, but it is still necessary to split TSSaRNA and cognate gene signals. To isolate the TSSaRNA signal, the most abundant read smaller than 151 bp is defined as the TSSaRNA full-length sequence. All other reads starting at the same position are related to the cognate gene. To be conservative, TSSaRNA reads are only retained if they sum up at least 10 counts.

### dRNA-seq library preparation, sequencing and analysis

Total RNAs were treated with Turbo™ DNase (Ambion) and incubated with Terminator™ 5′-phosphate-dependent exonuclease (Epicentre) (TEX+ sample) or only in buffer reaction (TEX- sample) at 30°C for 60 min, at proportion of 1 U TEX per 1 µg total RNA. Reaction products were purified with RNeasy MinElute Cleanup Kit (QIAGEN) and incubated with 1 U of Tobacco Acid Pyrophosphatase (TAP) (Epicentre) at 37°C for 1 hour in order to generate 5′-mono-phosphates RNAs able to bind to sequencing adapters. Reactions were purified again with RNeasy MinElute Cleanup Kit (QIAGEN).

Sequencing libraries were prepared with 1 µg of treated (TEX+) and untreated (TEX-) samples using a similar protocol described above for RNA-seq experiments. To ensure sequencing of a wider range of transcripts we increased the extension time on cDNA amplification step to 1 min and isolated molecules from 20 bp up to ∼480 bp on the gel. Paired-end sequencing was performed on Illumina Miseq v2 platform using 300 cycles kit. Forward reads were trimmed and mapped to the reference genome using Bowtie [32] as previous described. 435,339 reads corresponding to TSSaRNAs were used in subsequent analysis. TSSaRNAs presenting at least a 95% reads enrichment in TEX+ library relative to the TEX- library were considered as primary transcripts.

### Northern-blot

For Northern-blot analyzes, 30 µg of total RNA treated with RNAse-free DNAseI (Fermentas) was separated on polyacrylamide gel (8% acrylamide:bisacrylamide [29∶1], 8 M urea, 1xTris–borate–EDTA buffer). RNAs were transferred to Hybond-N+ membranes (GE Healthcare) and hybridized with ^32^P-labeled oligonucleotides (5′-AGTGTCGTTGAAGAAGTCAACTTCGCCTGTCGCCATTGCAACT-3′ for VNG0101G and 5′-AAAAGTGGCCGTGGGCAGCGGCCACCCGAT-3′ for VNG1213C) using Rapid-hyb buffer (GE Healthcare). Signals were detected by autoradiography using a M35A X-Omat Processor (Kodak). Genes encoding a conserved cold-shock protein (VNG0101G) (updated annotation: Supplementary Material 2 table from [14] and a probable exonuclease(VNG1213C) (updated annotation: UCSC Archaeal Genome Browser [24] and HaloLex project [33]) were chosen for this analysis.

### Promoter and structural analysis of TSSaRNA sequences

DNA sequences of 11 bp around TSSaRNA 3′ ends were analyzed for conserved patterns using MEME with default parameters [34] in order to identify possible RNA polymerase pausing site motifs. Secondary structures of TSSaRNAs were predicted using the GeneRfold Bioconductor package interface for Vienna RNA library [35]. In this analysis, Gibbs Free Energy of predicted structures derived from TSSaRNAs sequences were compared to sequences from non-cognate genes derived from similar regions.

### TSSaRNAs differential expression analysis

Differential expression of TSSaRNAs in *H. salinarum* NRC-1 was computed from a published dataset generated by tiling array hybridization of total RNA from 13 time points over a growth curve [14]. Using TSSaRNAs sequence coordinates information defined by single-base resolution RNA-seq, we revisited hybridization data and automatically selected a tiling array probe that best fits each TSSaRNA. The selected probe was required to have the highest TSSaRNA sequence coverage and, at the same time, should not cover any length beyond the TSSaRNA end (**Figure S10**). We compared the TSSaRNA representative probe intensity with the median intensity of the upstream region and also, with the intensity of the cognate gene. To be considered differentially expressed, this probe must have a substantial difference in relative intensity when compared to the other cognate gene probes and its surrounding (**Figure S11**). A TSSaRNA probe must show at least 10-fold difference relative to the overall relative intensity of its cognate gene: V = M_TSSaRNA_ – M_cognate_≥1, where M = log_10_(*t*/*t*_ref_), *t*_ref_ is taken at the reference time point in [14], *t* is taken at the growth curve time point when the second most different |V| is seen, M_cognate_ is the median of all cognate gene probes starting beyond TSSaRNA 3′ end. The same procedure is also required for an upstream region to make sure that TSSaRNA probe is not a merely continuum of adjacent transcript signal. Therefore, a differentially expressed probe must also show at least a 2-fold difference relative to the overall relative intensity of an upstream region. This upstream region is 300 bp long, 120 bp away from TSSaRNA start (**Figure S10**). If, there is an annotated gene closer than 200 bp from the TSSaRNA start, the aforementioned region is ignored and the whole adjacent CDS region is considered for probe averaging.

### RNA polymerase pausing computational model

A simple RNA polymerase pausing model was created (**Figure S6**) and implemented in R programing language (**File S1, File S2**). The model attributes a waiting time for each base position along a virtual gene. For simplicity, this waiting time is taken to be 1 arbitrary time unit. A RNA polymerase pausing site is a position where a moving RNA polymerase stalls for more than the default waiting time. This time is called “stalled time” (Δ*t*). There is an “intrinsic transcription initiation time interval” (Δ*τ*), which is the time it takes between two successive RNA polymerases to start their trajectory along the gene from the first base pair to the gene's end. These two time interval parameters are the most important parameters. Other auxiliary parameters are: gene length *L*, pause position *L′* and total simulation elapsed time *T*. A RNA polymerase is not allowed to keep traveling along the gene if there is another one stalled at the next base pair. In this case it releases its transcript and detaches from DNA, terminating the transcription process. Also, the stalled RNA polymerase that blocked the previous one is not affected and only keep moving forward when its waiting time at pausing site is up.

## Supporting Information

Figure S1 **Data-mining on archaeal gene expression public datasets.** For all archaea, the yellow arrow represents the cognate gene, blue dashed lines represent TSSaRNA estimated regions and horizontal axis represent organism's genome coordinates. Heatmaps for **A, B, C** and **D** represent gene expression profiles over growth curves. Heatmaps are color-coded according to log10 expression ratios between each time point relative to reference growth condition samples. Light blue horizontal bars for **A, B, C** represent tiling array probe intensities for reference conditions. Dark blue points for **D, E, F, G** and **H** represent RNA-seq reads coverage data. Frames **I** and **J** were extracted directly, with minor adjustments, from published figures. Reads in **I** were originally from Jäger et al 2009's Figure 1. Reads in **J** were originally from Toffano-Nioche et al 2013's Figure S4. Light blue crosses for **K** and **L** represent Nimblegen tiling array probe intensities for the reference conditions. Red crosses for **K** and **L** represent Nimblegen tiling array probe which best matches the TSSaRNA.
(PDF)

Figure S2 **Additional examples of transcriptome mapping using small RNA sequencing.** Strand-specific RNA-seq experiment was performed using non-fragmented small RNAs (20–230 pb). Panels A–D show examples of TSSaRNAs and their cognate genes. (A) VNG0725H, forward strand (B) VNG1182H, reverse strand (C) VNG2014H, forward strand. (D) VNG2658G, reverse strand. For each panel, the genome position and CDSs location and orientation (grey arrows) are indicated at the horizontal axis. The uppermost graphic show log2 transformed amount of reads covering a given genomic coordinate. Vertical dashed lines represents the TSSaRNA region. The intermediary graphic represents the amount of aligned reads whose start position maps to a given genomic coordinate, the “start profile”.
(PDF)

Figure S3 **Properties of the 179 TSSaRNAs identified by small RNA-seq and dRNA-seq.** **A** – Size distribution. **B** – Distribution of the distances between TSSaRNA start position and cognate gene CDSs start codon position. **C** – Distribution of Pearson correlation between each TSSaRNA and its cognate gene.
(PDF)

Figure S4 **Schematic illustration of a putative signature if non-degradative processing biogenesis hypotheses would hold.** Dark blue points represent RNA-seq reads coverage data. The yellow arrow represents a gene. Green vertical bars represent mapped reads start positions along genome coordinates and their abundances. Light blue highlight represents TSSaRNA sequence region. The prediction illustrated by the figure **is not** found in *H. salinarum* sequencing experiments.
(PDF)

Figure S5 **Gibbs Free Energy distribution of secondary structure predictions for TSSaRNAs and regular near-TSS sequences.** Histogram considers non-cognate genes sequences with similar localization and same size as the TSSaRNA median sizes. Vertical blue bars represent values for actual TSSaRNA sequences.
(PDF)

Figure S6 **Schematic illustration of the RNA polymerase pausing computational model.** An arbitrary gene of length *L* bp is considered. Genomic position *L′* represents the pausing site. Each moment in time is depicted by successive drawings from the upper left panel downwards until the rightmost lower panel. Time passing is not represented in constant flux and downward vertical arrows illustrate the amount of time passed. Every Δ*τ* units of time a new RNA polymerase arrives at position 1 bp and keep transcribing forward at a constant velocity of *v* bp/unit of time. Arriving at the pausing point, a RNA polymerase receives *v* = 0 and waits there for Δ*t* units of time, leaving then again with the same velocity *v* until it reaches the end of the gene at position *L* and releasing the full-length transcript. The parameters Δ*τ* and Δ*t* are the most critical for the model and are called “time spent stalled” and “intrinsic transcription initiation interval”, respectively. If an incoming RNA polymerase encounter another RNA polymerase just a base pair downstream, it cannot go further and releases the DNA sequence freeing the transcript synthesized up to that position/moment. The RNA polymerases released due to downstream blocking are shown with their IDs inside their red circle representation and those still active are show with their IDs below. This illustration depicts several moments between the first RNA polymerase (ID #1) start at position 1 bp until it reaches the last position *L* bp, along with several RNA polymerases (IDs #2, #3, #5, …, #n+2) that produced TSSaRNAs due to early transcription termination.
(PDF)

Figure S7 **Expression profiles of TSSaRNAs differentially expressed over a typical growth-curve relative to the control growth condition.** Horizontal axis represent 13 growth curve points from different phases in a standard laboratory batch culture. Solid lines are TSSaRNA expression profiles and dashed lines their cognate gene expression profile.
(PDF)

Figure S8 **Simple RNA polymerase pausing computational model simulation.** Simulated expression profiles of TSSaRNAs differentially expressed during a growth curve relative to the control condition. Tiling microarray output simulation for a 35 bp TSSaRNA in a 2 kb cognate gene. Varying the only two model parameters it is possible to generate situations in which the cognate gene expression level remains constant over time and TSSaRNA levels can vary almost arbitrarily. Panels A to D are build mimicking our experimental setup displayed in manuscript's Figures 4 and Figure S7 exploring the parameter space. Vertical-axis – log2 ratios between simulated quantity of transcripts in each time point (*I_t_*) and the amount simulated at reference time-point (*I_ref_*). Black solid line – TSSaRNA expression profile. Black dashed line – cognate gene expression profile (constant over time and arbitrarily set to the same value of reference condition). Panel E shows all kinds of log2 rations that can be obtained for a TSSaRNA probe and its cognate gene when scanning the parameter space: Δ*τ* and Δ*t*, “intrinsic transcription initiation interval” and “time spent stalled”, respectively. This example scans Δ*τ* from 2 to 700 time units, Δ*t* from 3 to 700 time units and simulates a 2 kb gene with a 35 bp TSSaRNA associated. Highlighted points in Panel E are examples of relatively constant TSSaRNA levels with an appropriately 3-fold difference in cognate gene level (light blue and purple circles, corresponding to Δ*t* = 46 and Δ*τ* = 12 time units and Δ*t* = 14 and Δ*τ* = 8 time units, respectively), and a 32-fold difference in TSSaRNA levels with relatively constant cognate gene levels (red and green circles, corresponding to Δ*t* = 250 and Δ*τ* = 4 time units and Δ*t* = 250 and Δ*τ* = 84 time units, respectively). Qualitatively, almost any complex dynamical behavior can be obtained if the pausing rhythm and the RNA polymerase arriving rate are jointly regulated by environmental clues.
(PDF)

Figure S9 **Illustration of the TSSaRNA identification procedure.** The yellow arrow represents a cognate gene's coding sequence region (CDS). Green vertical bars represent mapped reads' start coordinates and their abundances. The grey circle highlights the most frequent start coordinate. The grey box zoom illustrates the set of reads which mapped to this specific coordinate position, composed by two populations: identical reads from TSSaRNAs and other reads that originate from cognate gene transcripts. Black horizontal bars represent regions, relative to translation initiation site position (start codon position), around which the search for the most frequent start coordinate position was performed.
(PDF)

Figure S10 **Illustration of the probe selection for TSSaRNA differential expression analysis.** Light blue horizontal bars illustrate tiling array probe intensities for the reference condition. The best probe that represent the TSSaRNA is highlighted in red. The yellow arrow represents a cognate gene. Black dashed lines represent the TSSaRNA boundaries defined by RNA-seq. Gray boxes represent the regions used to calculate the neighbourhood expression intensity, which was compared to the TSSaRNA probe.
(PDF)

Figure S11 **Illustration of the method used to define a differentially expressed TSSaRNA in *H. salinarum*.** The three-dimensional tiling microarray data (relative intensities vs growth-curve vs genome loci) is reduced to two dimensions and then to a single representative value. A TSSaRNA expression profile is considered distinct from its cognate gene if there are at least two time-points in which their relative intensities are at least 10-fold apart. Relative intensities are considered between a time-point and the reference growth condition. Relative intensities for TSSaRNAs are provided by the best tiling array probe (red horizontal bar) to fit a RNA-seq based TSSaRNA boundaries definition (see Figure S10). Relative intensities for their cognate genes are provided by the median of all non-overlaping adjacent tiling array probes (horizontal magenta dashed line). From all time-points along the growth-curve, the final differential expression value to be reported is from the data slice (fold-change vs position projection) where the 2^nd^ top difference between TSSaRNA and cognate gene is found (blue vertical dashed line at *t* = 5).
(PDF)

Table S1 TSSaRNAs identified in *Halobacterium salinarum* transcriptome.
(XLS)

Table S2 Subset of TSSaRNAs identified in *Halobacterium salinarum* transcriptome differentially regulated relative to their cognate gene.
(XLS)

Table S3 Detailed information on all 10 organism datasets used to detect TSSaRNA in archaea.
(XLS)

File S1 **RNApol pausing computational model with multiple pausing sites (R language script).**
(R) Click here for additional data file.

File S2 **RNApol pausing computational model with varying pausing rhythm (R language script).**
(R) Click here for additional data file.
