# Supplementary material for: Transcription Start Site Associated RNAs (TSSaRNAs) Are Ubiquitous in All Domains of Life
Source: PLoS One. 2014 Sep 19;9(9):e107680. doi: 10.1371/journal.pone.0107680 (PMC4169567; doi:10.1371/journal.pone.0107680)
Supplement: Figure S1 — Data-mining on archaeal gene expression public datasets. For all archaea, the yellow arrow represents the cognate gene, blue dashed lines represent TSSaRNA estimated regions and horizontal axis represent organism's genome coordinates. Heatmaps for A, B, C and D represent gene expression profiles over growth curves. Heatmaps are color-coded according to log10 expression ratios between each time point relative to reference growth condition samples. Light blue horizontal bars for A, B, C represent tiling array probe intensities for reference conditions. Dark blue points for D, E, F, G and H represent RNA-seq reads coverage data. Frames I and J were extracted directly, with minor adjustments, from published figures. Reads in I were originally from Jäger et al 2009's Figure 1. Reads in J were originally from Toffano-Nioche et al 2013's Figure S4. Light blue crosses for K and L represent Nimblegen tiling array probe intensities for the reference conditions. Red crosses for K and L represent Nimblegen tiling array probe which best matches the TSSaRNA. (PDF) [file pone.0107680.s001.pdf]

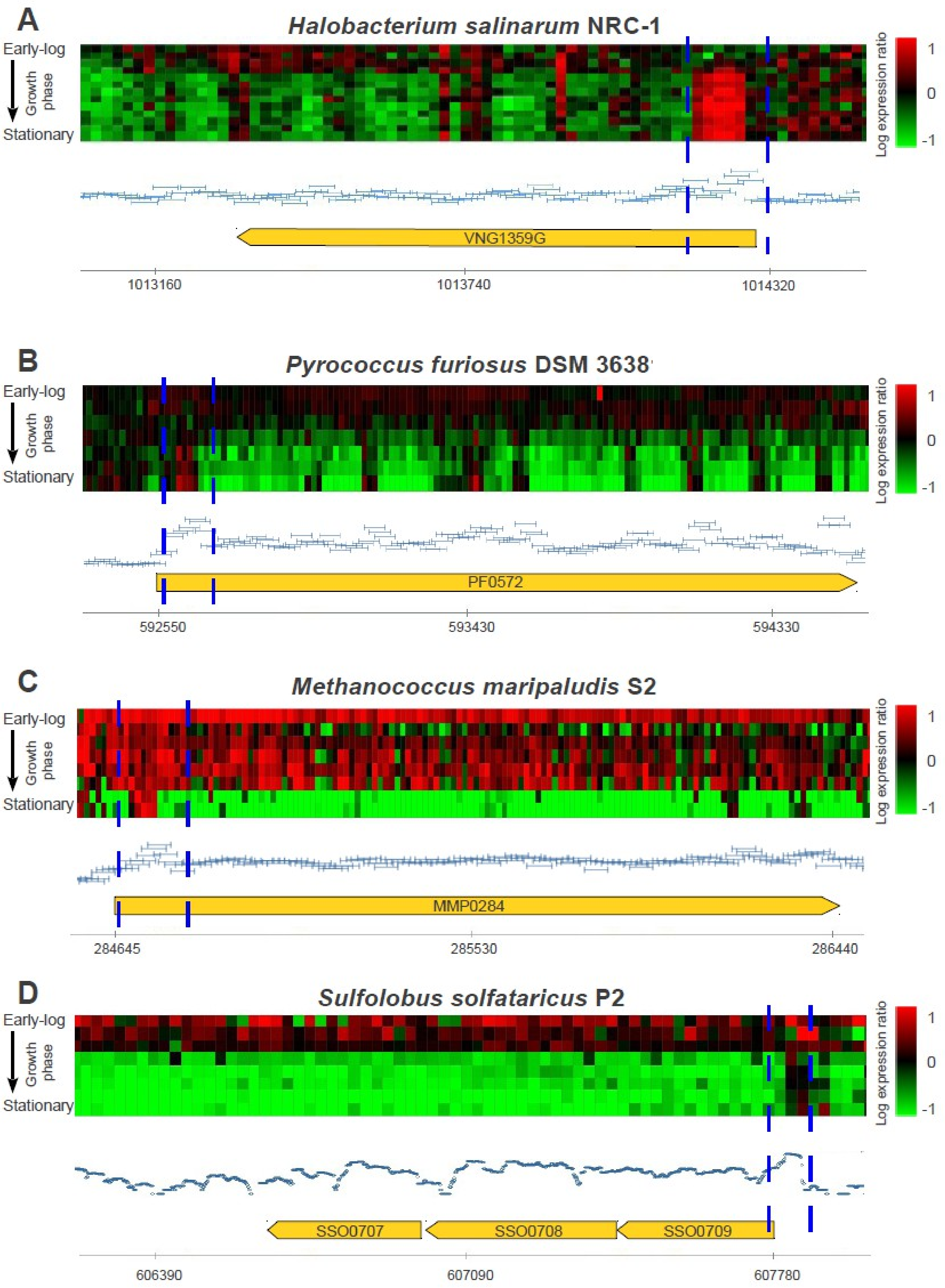
A
Halobacterium salinarum NRC-1
Early-log
Growth phase
Stationary
Log expression ratio
1
0
-1
VNG1359G
1013160
1013740
1014320
B
Pyrococcus furiosus DSM 3638
Early-log
Growth phase
Stationary
Log expression ratio
1
0
-1
PF0572
592550
593430
594330
C
Methanococcus maripaludis S2
Early-log
Growth phase
Stationary
Log expression ratio
1
0
-1
MMP0284
284645
285530
286440
D
Sulfolobus solfataricus P2
Early-log
Growth phase
Stationary
Log expression ratio
1
0
-1
SSO0707
SSO0708
SSO0709
606390
607090
607780

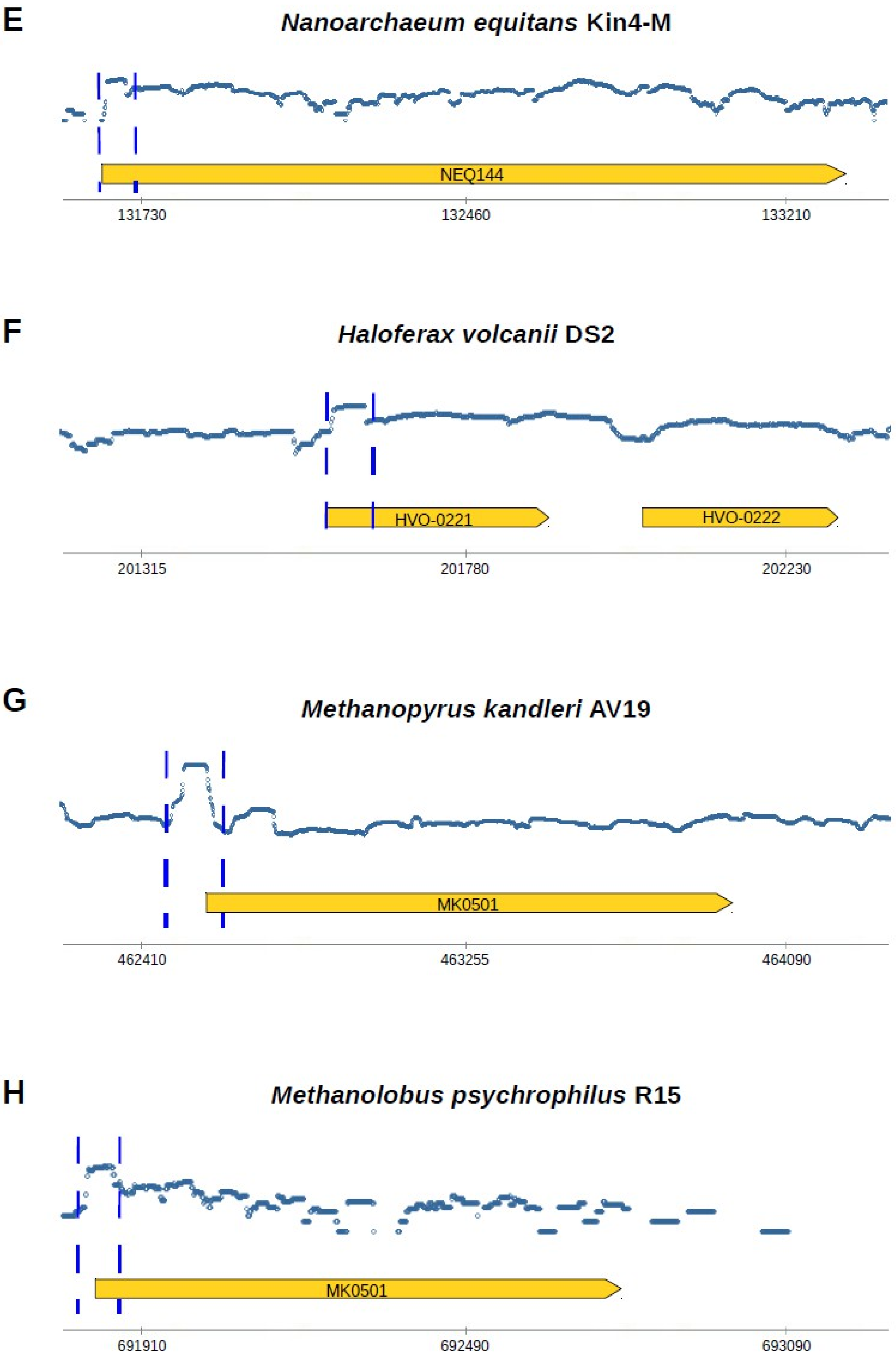

E
Nanoarchaeum equitans Kin4-M
NEQ144
131730
132460
133210
F
Haloferax volcanii DS2
HVO-0221
HVO-0222
201315
201780
202230
G
Methanopyrus kandleri AV19
MK0501
462410
463255
464090
H
Methanolobus psychrophilus R15
MK0501
691910
692490
693090

Halobacterium salinarum NRC-1

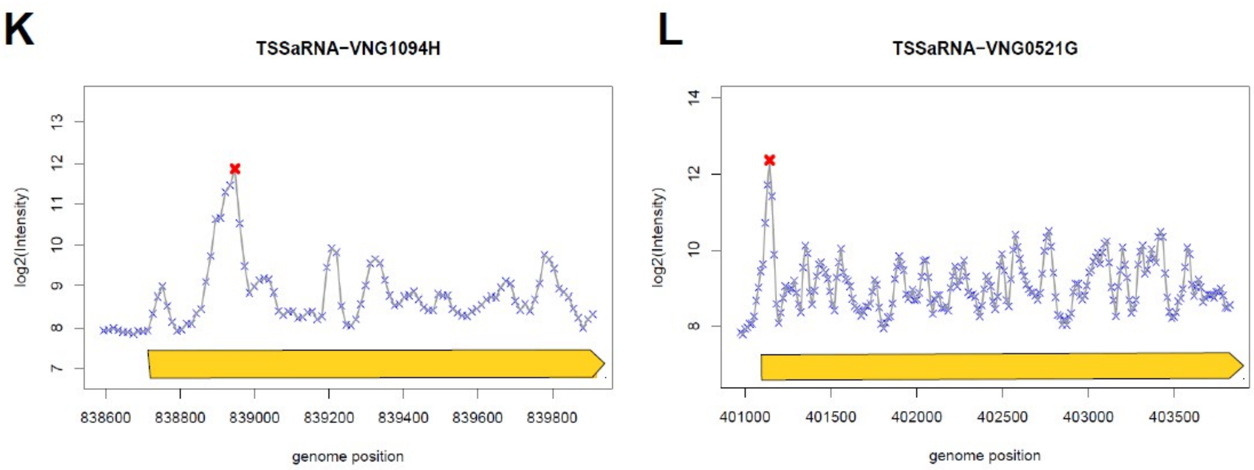

Figure S1 – Data-mining on archaeal gene expression public datasets. For all archaea, the yellow arrow represents the cognate gene, blue dashed lines represent TSSaRNA estimated regions and horizontal axis represent organism's genome coordinates. Heatmaps for **A**, **B**, **C** and **D** represent gene expression profiles over growth curves. Heatmaps are color-coded according to log10 expression ratios between each time point relative to reference growth condition samples. Light blue horizontal bars for **A**, **B**, **C** represent tiling array probe intensities for reference conditions. Dark blue points for **D**, **E**, **F**, **G** and **H** represent RNA-seq reads coverage data. Frames **I** and **J** were extracted directly, with minor adjustments, from published figures. Reads in **I** were originally from Jäger et al 2009's Figure 1. Reads in **J** were originally from Toffano-Nioche et al 2013's Supplemental Material Figure S4. Light blue crosses for **K** and **L** represent Nimblegen tiling array probe intensities for the reference conditions. Red crosses for **K** and **L** represent Nimblegen tiling array probe which best matches the TSSaRNA.
