# Supplementary material for: Transcription Start Site Associated RNAs (TSSaRNAs) Are Ubiquitous in All Domains of Life
Source: PLoS One. 2014 Sep 19;9(9):e107680. doi: 10.1371/journal.pone.0107680 (PMC4169567; doi:10.1371/journal.pone.0107680)
Supplement: Figure S2 — Additional examples of transcriptome mapping using small RNA sequencing. Strand-specific RNA-seq experiment was performed using non-fragmented small RNAs (20–230 pb). Panels A–D show examples of TSSaRNAs and their cognate genes. (A) VNG0725H, forward strand (B) VNG1182H, reverse strand (C) VNG2014H, forward strand. (D) VNG2658G, reverse strand. For each panel, the genome position and CDSs location and orientation (grey arrows) are indicated at the horizontal axis. The uppermost graphic show log2 transformed amount of reads covering a given genomic coordinate. Vertical dashed lines represents the TSSaRNA region. The intermediary graphic represents the amount of aligned reads whose start position maps to a given genomic coordinate, the “start profile”. (PDF) [file pone.0107680.s002.pdf]

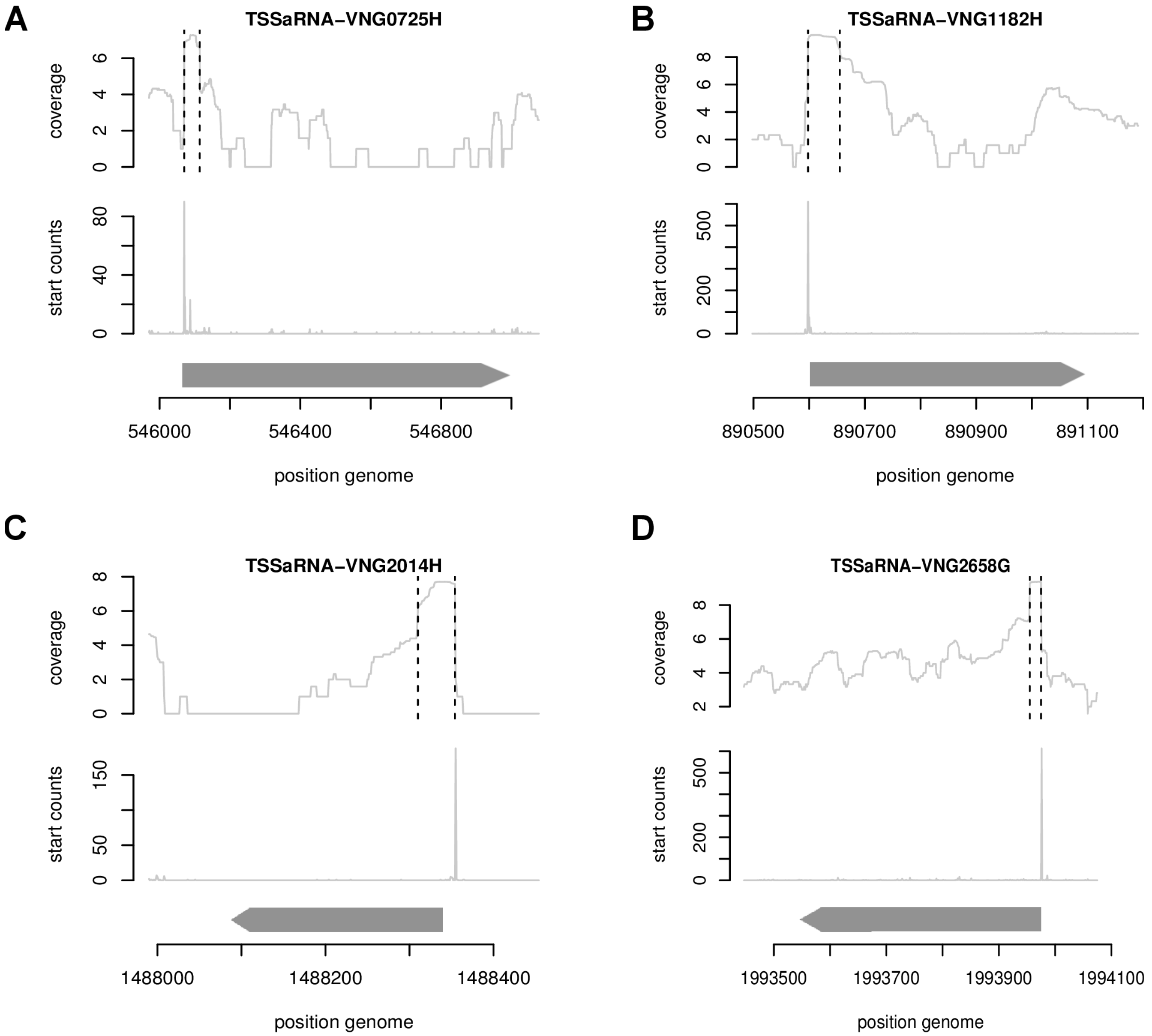

Figure S2 – Additional examples of transcriptome mapping using small RNA sequencing. Strand-specific RNA-seq experiment was performed using non-fragmented small RNAs (20-230pb). Panels A-D show examples of TSSaRNAs and their cognate genes. (A) VNG0725H, forward strand (B) VNG1182H, reverse strand (C) VNG2014H, forward strand. (D) VNG2658G, reverse strand. For each panel, the genome position and CDSs location and orientation (grey arrows) are indicated at the horizontal axis. The uppermost graphic show log2 transformed amount of reads covering a given genomic coordinate. Vertical dashed lines represents the TSSaRNA region. The intermediary graphic represents the amount of aligned reads whose start position maps to a given genomic coordinate, the “start profile”.
