# Supplementary material for: Transcription Start Site Associated RNAs (TSSaRNAs) Are Ubiquitous in All Domains of Life
Source: PLoS One. 2014 Sep 19;9(9):e107680. doi: 10.1371/journal.pone.0107680 (PMC4169567; doi:10.1371/journal.pone.0107680)
Supplement: Figure S3 — Properties of the 179 TSSaRNAs identified by small RNA-seq and dRNA-seq. A – Size distribution. B – Distribution of the distances between TSSaRNA start position and cognate gene CDSs start codon position. C – Distribution of Pearson correlation between each TSSaRNA and its cognate gene. (PDF) [file pone.0107680.s003.pdf]

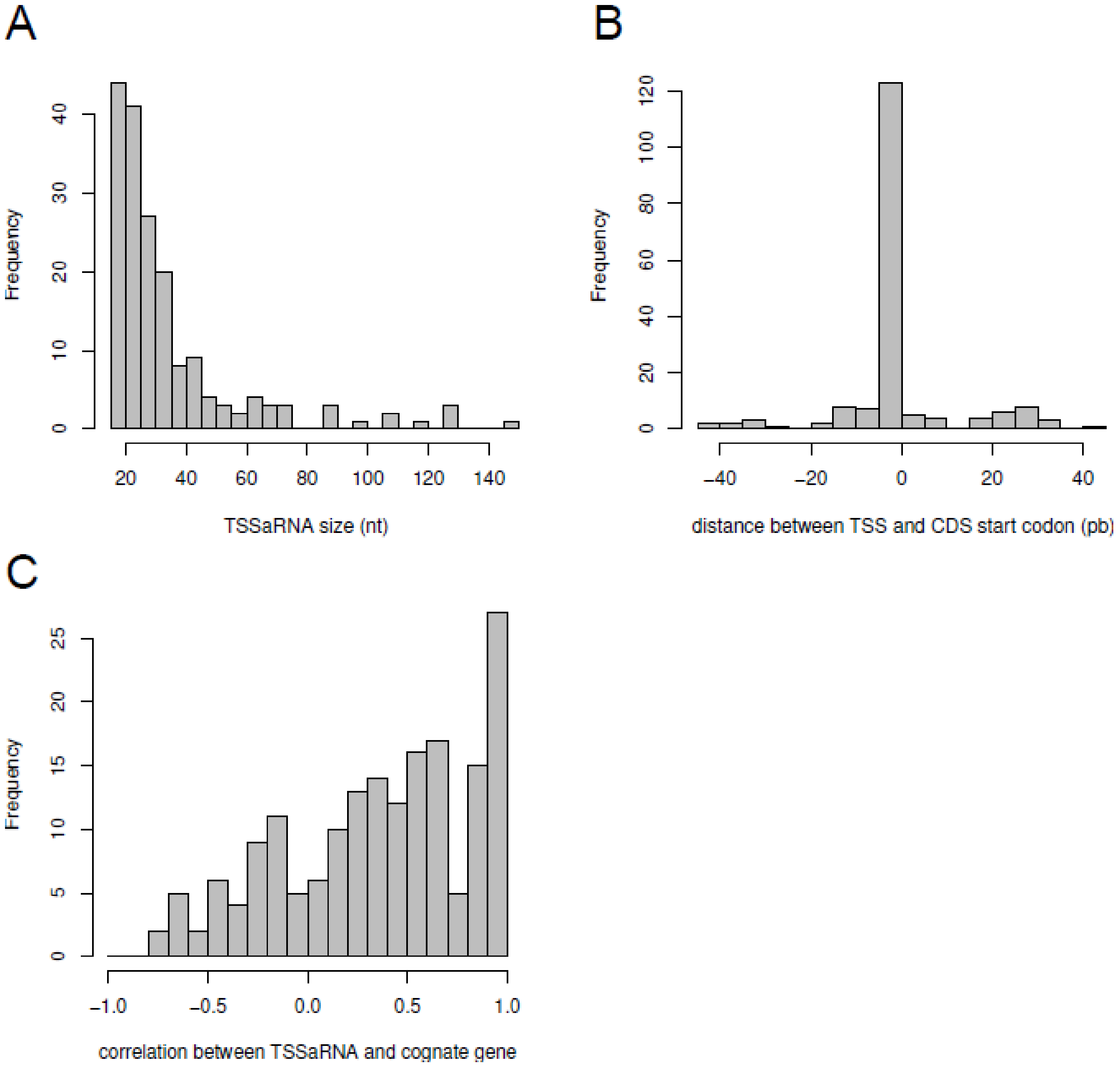

Figure S3 – Properties of the 179 TSSaRNAs identified by small RNA-seq and dRNA-seq. A – Size distribution. **B** – Distribution of the distances between TSSaRNA start position and cognate gene CDSs start codon position. **C** – Distribution of Pearson correlation between each TSSaRNA and its cognate gene.
