# Supplementary material for: Transcription Start Site Associated RNAs (TSSaRNAs) Are Ubiquitous in All Domains of Life
Source: PLoS One. 2014 Sep 19;9(9):e107680. doi: 10.1371/journal.pone.0107680 (PMC4169567; doi:10.1371/journal.pone.0107680)
Supplement: Figure S4 — Schematic illustration of a putative signature if non-degradative processing biogenesis hypotheses would hold. Dark blue points represent RNA-seq reads coverage data. The yellow arrow represents a gene. Green vertical bars represent mapped reads start positions along genome coordinates and their abundances. Light blue highlight represents TSSaRNA sequence region. The prediction illustrated by the figure is not found in H. salinarum sequencing experiments. (PDF) [file pone.0107680.s004.pdf]

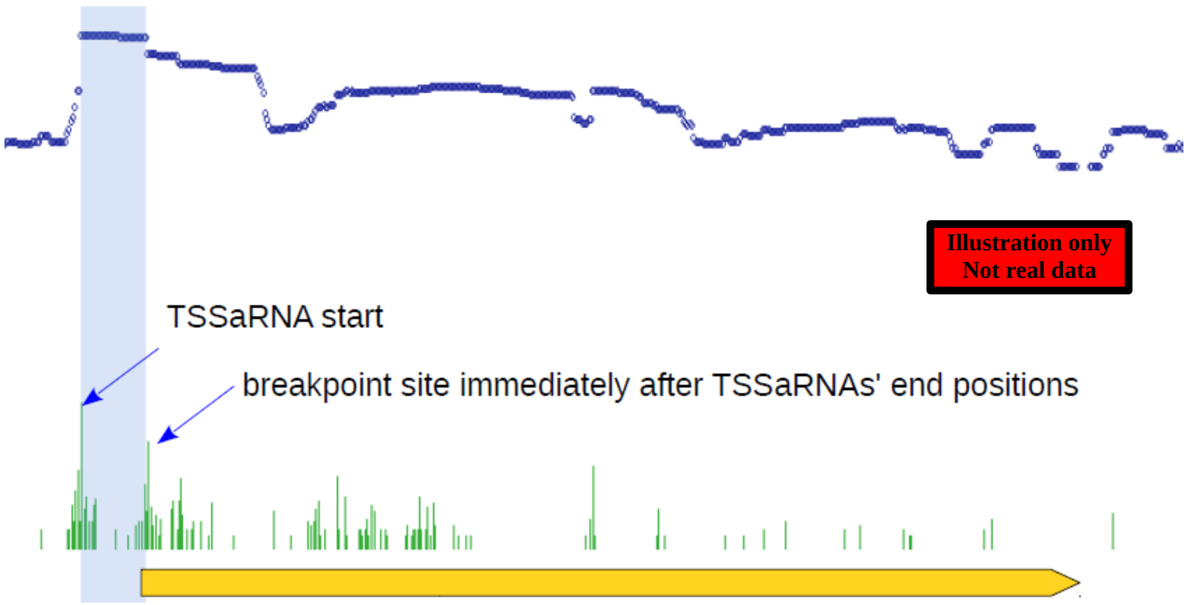

Figure S4 – Schematic illustration of a putative signature if non-degradative processing biogenesis hypotheses would hold. Dark blue points represent RNA-seq reads coverage data. The yellow arrow represents a gene. Green vertical bars represent mapped reads start positions along genome coordinates and their abundances. Light blue highlight represents TSSaRNA sequence region. The prediction illustrated by the figure **is not** found in *H.salinarum* sequencing experiments.
