# Supplementary material for: Transcription Start Site Associated RNAs (TSSaRNAs) Are Ubiquitous in All Domains of Life
Source: PLoS One. 2014 Sep 19;9(9):e107680. doi: 10.1371/journal.pone.0107680 (PMC4169567; doi:10.1371/journal.pone.0107680)
Supplement: Figure S5 — Gibbs Free Energy distribution of secondary structure predictions for TSSaRNAs and regular near-TSS sequences. Histogram considers non-cognate genes sequences with similar localization and same size as the TSSaRNA median sizes. Vertical blue bars represent values for actual TSSaRNA sequences. (PDF) [file pone.0107680.s005.pdf]

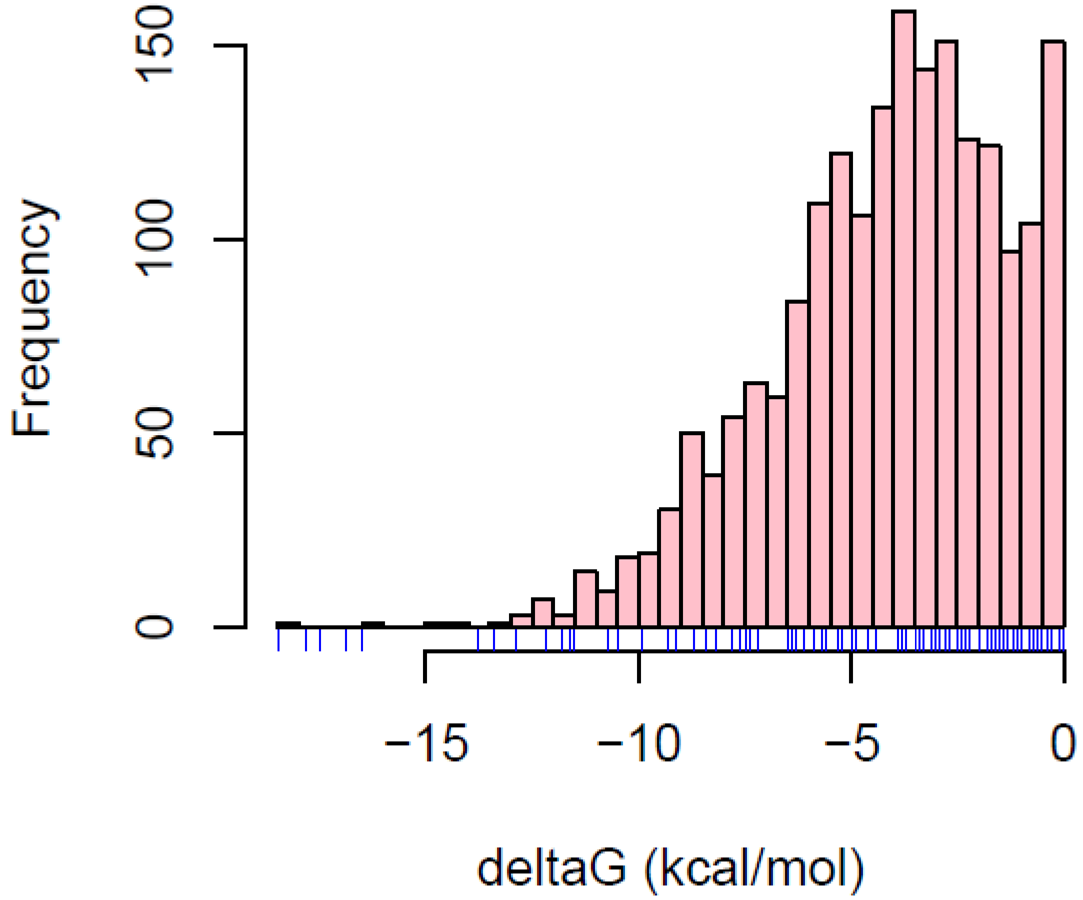

Figure S5 – Gibbs Free Energy distribution of secondary structure predictions for TSSaRNAs and regular near-TSS sequences. Histogram considers non-cognate genes sequences with similar localization and same size as the TSSaRNA median sizes. Vertical blue bars represent values for actual TSSaRNA sequences.
