# Supplementary material for: Transcription Start Site Associated RNAs (TSSaRNAs) Are Ubiquitous in All Domains of Life
Source: PLoS One. 2014 Sep 19;9(9):e107680. doi: 10.1371/journal.pone.0107680 (PMC4169567; doi:10.1371/journal.pone.0107680)
Supplement: Figure S6 — Schematic illustration of the RNA polymerase pausing computational model. An arbitrary gene of length L bp is considered. Genomic position L′ represents the pausing site. Each moment in time is depicted by successive drawings from the upper left panel downwards until the rightmost lower panel. Time passing is not represented in constant flux and downward vertical arrows illustrate the amount of time passed. Every Δτ units of time a new RNA polymerase arrives at position 1 bp and keep transcribing forward at a constant velocity of v bp/unit of time. Arriving at the pausing point, a RNA polymerase receives v = 0 and waits there for Δt units of time, leaving then again with the same velocity v until it reaches the end of the gene at position L and releasing the full-length transcript. The parameters Δτ and Δt are the most critical for the model and are called “time spent stalled” and “intrinsic transcription initiation interval”, respectively. If an incoming RNA polymerase encounter another RNA polymerase just a base pair downstream, it cannot go further and releases the DNA sequence freeing the transcript synthesized up to that position/moment. The RNA polymerases released due to downstream blocking are shown with their IDs inside their red circle representation and those still active are show with their IDs below. This illustration depicts several moments between the first RNA polymerase (ID #1) start at position 1 bp until it reaches the last position L bp, along with several RNA polymerases (IDs #2, #3, #5, …, #n+2) that produced TSSaRNAs due to early transcription termination. (PDF) [file pone.0107680.s006.pdf]

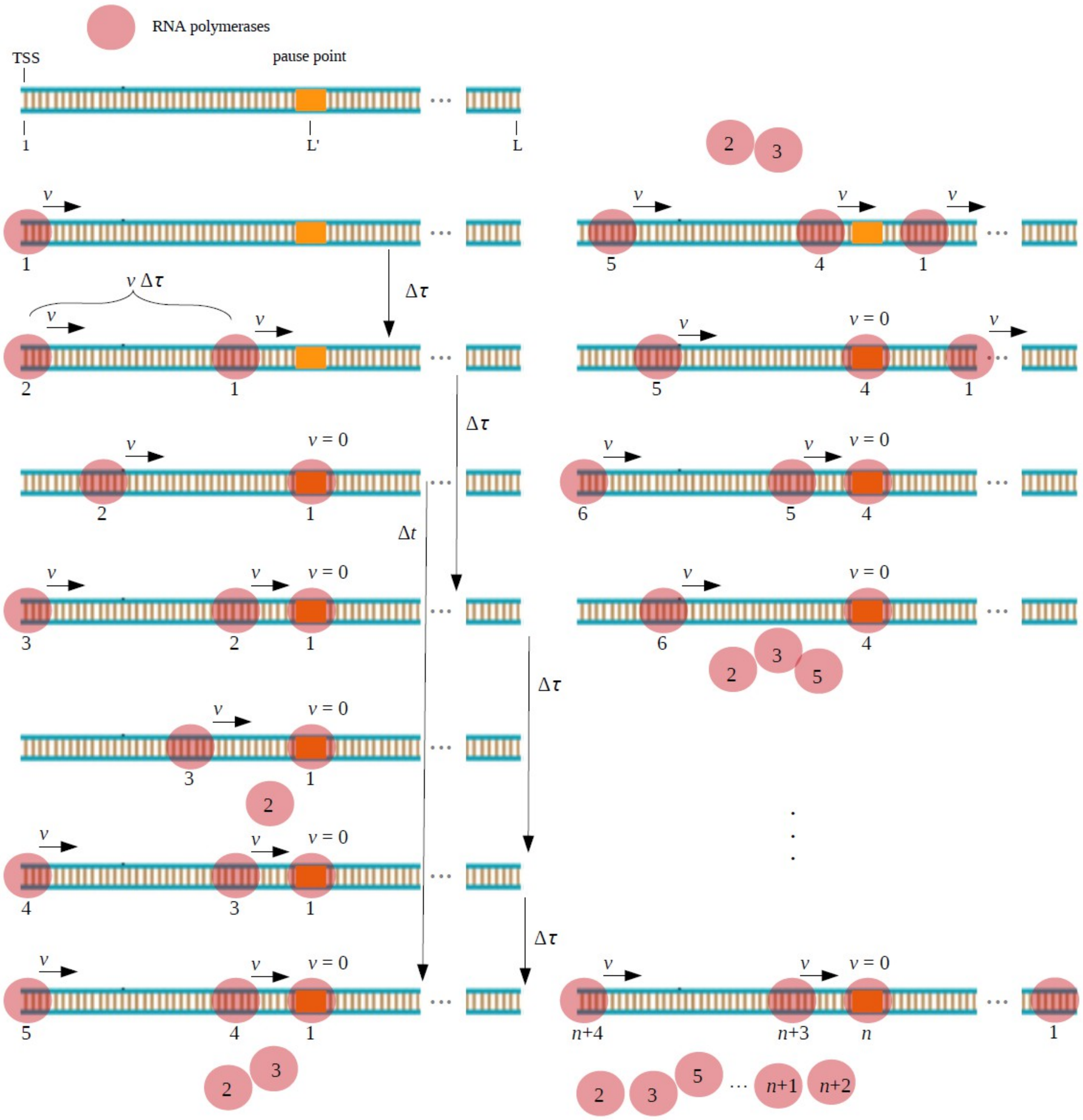


**Figure S6 – Schematic illustration of the RNA polymerase pausing computational model.** An arbitrary gene of length $L$ bp is considered. Genomic position $L'$ represents the pausing site. Each moment in time is depicted by successive drawings from the upper left panel downwards until the rightmost lower panel. Time passing is not represented in constant flux and downward vertical arrows illustrate the amount of time passed. Every $\Delta\tau$ units of time a new RNA polymerase arrives at position 1bp and keep transcribing forward at a constant velocity of $v$ bp/unit of time. Arriving at the pausing point, a RNA polymerase receives $v = 0$ and waits there for $\Delta t$ units of time, leaving then again with the same velocity $v$ until it reaches the end of the gene at position $L$ and releasing the full-length transcript. The parameters $\Delta\tau$ and $\Delta t$ are the most critical for the model and are called “time spent stalled” and “intrinsic transcription initiation interval”, respectively. If an incoming RNA polymerase encounter

another RNA polymerase just a base pair downstream, it cannot go further and releases the DNA sequence freeing the transcript synthesized up to that position/moment. The RNA polymerases released due to downstream blocking are shown with their IDs inside their red circle representation and those still active are show with their IDs below. This illustration depicts several moments between the first RNA polymerase (ID #1) start at position 1bp until it reaches the last position *L* bp, along with several RNA polymerases (IDs #2, #3, #5, …, #n+2) that produced TSSaRNAs due to early transcription termination.
