# Supplementary material for: Transcription Start Site Associated RNAs (TSSaRNAs) Are Ubiquitous in All Domains of Life
Source: PLoS One. 2014 Sep 19;9(9):e107680. doi: 10.1371/journal.pone.0107680 (PMC4169567; doi:10.1371/journal.pone.0107680)
Supplement: Figure S7 — Expression profiles of TSSaRNAs differentially expressed over a typical growth-curve relative to the control growth condition. Horizontal axis represent 13 growth curve points from different phases in a standard laboratory batch culture. Solid lines are TSSaRNA expression profiles and dashed lines their cognate gene expression profile. (PDF) [file pone.0107680.s007.pdf]

TSSaRNA-VNG0249G

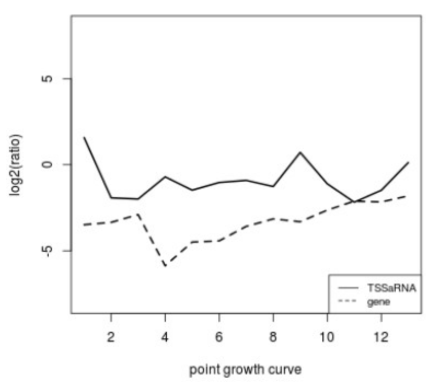

TSSaRNA-VNG0650C

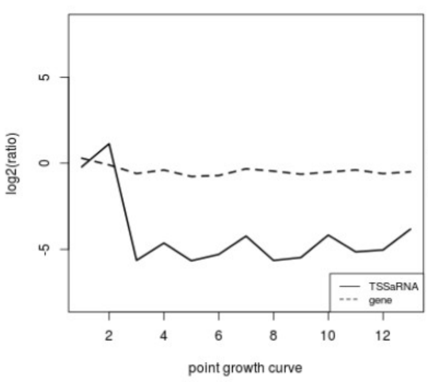

TSSaRNA-VNG2246H

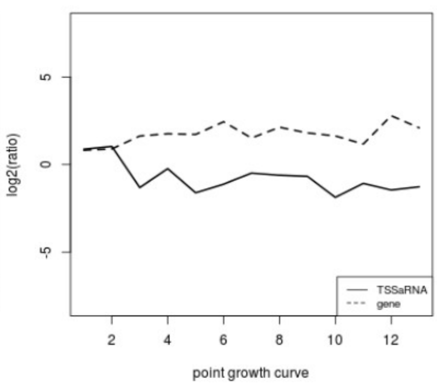

TSSaRNA-VNG0999H

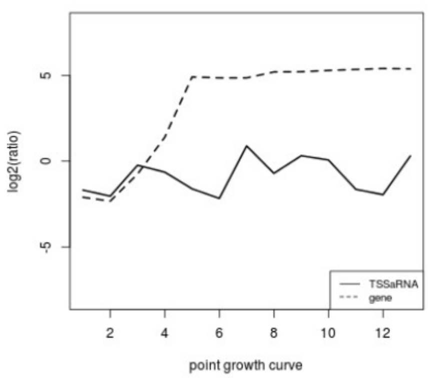

TSSaRNA-VNG1213C

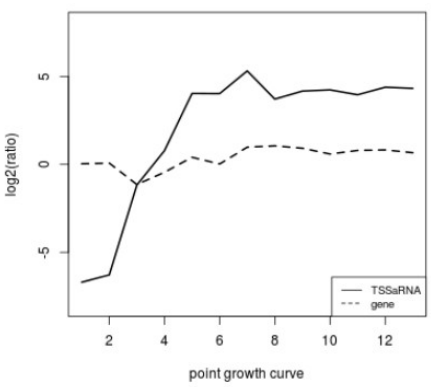

TSSaRNA-VNG1589C

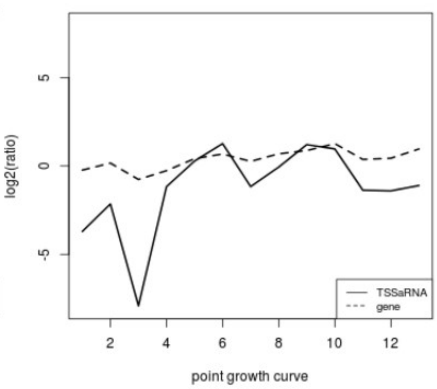

TSSaRNA-VNG2537G

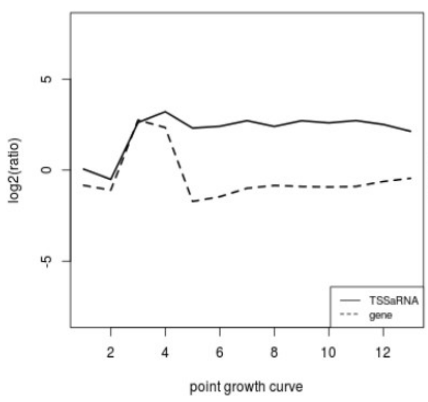

TSSaRNA-VNG1380H

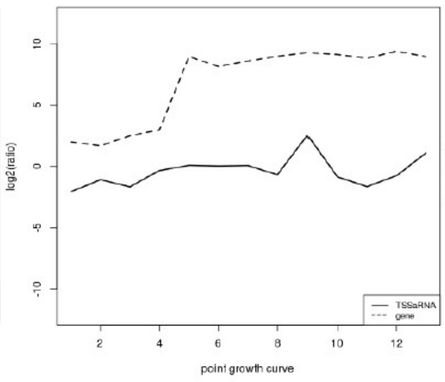

TSSaRNA-VNG2508C

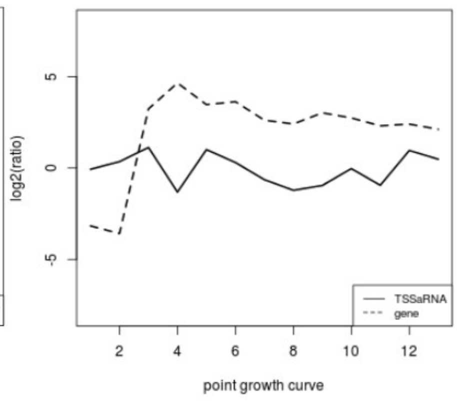

TSSaRNA-VNG6194H

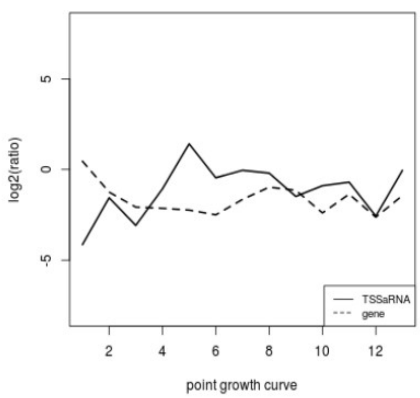

Figure S7 – Expression profiles of TSSaRNAs differentially expressed over a typical growth-curve relative to the control growth condition. Horizontal axis represent 13 growth curve points from different phases in a standard laboratory batch culture. Solid lines are TSSaRNA expression profiles and dashed lines their cognate gene expression profile.
