# Supplementary material for: Transcription Start Site Associated RNAs (TSSaRNAs) Are Ubiquitous in All Domains of Life
Source: PLoS One. 2014 Sep 19;9(9):e107680. doi: 10.1371/journal.pone.0107680 (PMC4169567; doi:10.1371/journal.pone.0107680)
Supplement: Figure S8 — Simple RNA polymerase pausing computational model simulation. Simulated expression profiles of TSSaRNAs differentially expressed during a growth curve relative to the control condition. Tiling microarray output simulation for a 35 bp TSSaRNA in a 2 kb cognate gene. Varying the only two model parameters it is possible to generate situations in which the cognate gene expression level remains constant over time and TSSaRNA levels can vary almost arbitrarily. Panels A to D are build mimicking our experimental setup displayed in manuscript's Figures 4 and Figure S7 exploring the parameter space. Vertical-axis – log2 ratios between simulated quantity of transcripts in each time point (It) and the amount simulated at reference time-point (Iref). Black solid line – TSSaRNA expression profile. Black dashed line – cognate gene expression profile (constant over time and arbitrarily set to the same value of reference condition). Panel E shows all kinds of log2 rations that can be obtained for a TSSaRNA probe and its cognate gene when scanning the parameter space: Δτ and Δt, “intrinsic transcription initiation interval” and “time spent stalled”, respectively. This example scans Δτ from 2 to 700 time units, Δt from 3 to 700 time units and simulates a 2 kb gene with a 35 bp TSSaRNA associated. Highlighted points in Panel E are examples of relatively constant TSSaRNA levels with an appropriately 3-fold difference in cognate gene level (light blue and purple circles, corresponding to Δt = 46 and Δτ = 12 time units and Δt = 14 and Δτ = 8 time units, respectively), and a 32-fold difference in TSSaRNA levels with relatively constant cognate gene levels (red and green circles, corresponding to Δt = 250 and Δτ = 4 time units and Δt = 250 and Δτ = 84 time units, respectively). Qualitatively, almost any complex dynamical behavior can be obtained if the pausing rhythm and the RNA polymerase arriving rate are jointly regulated by environmental clues. (PDF) [file pone.0107680.s008.pdf]

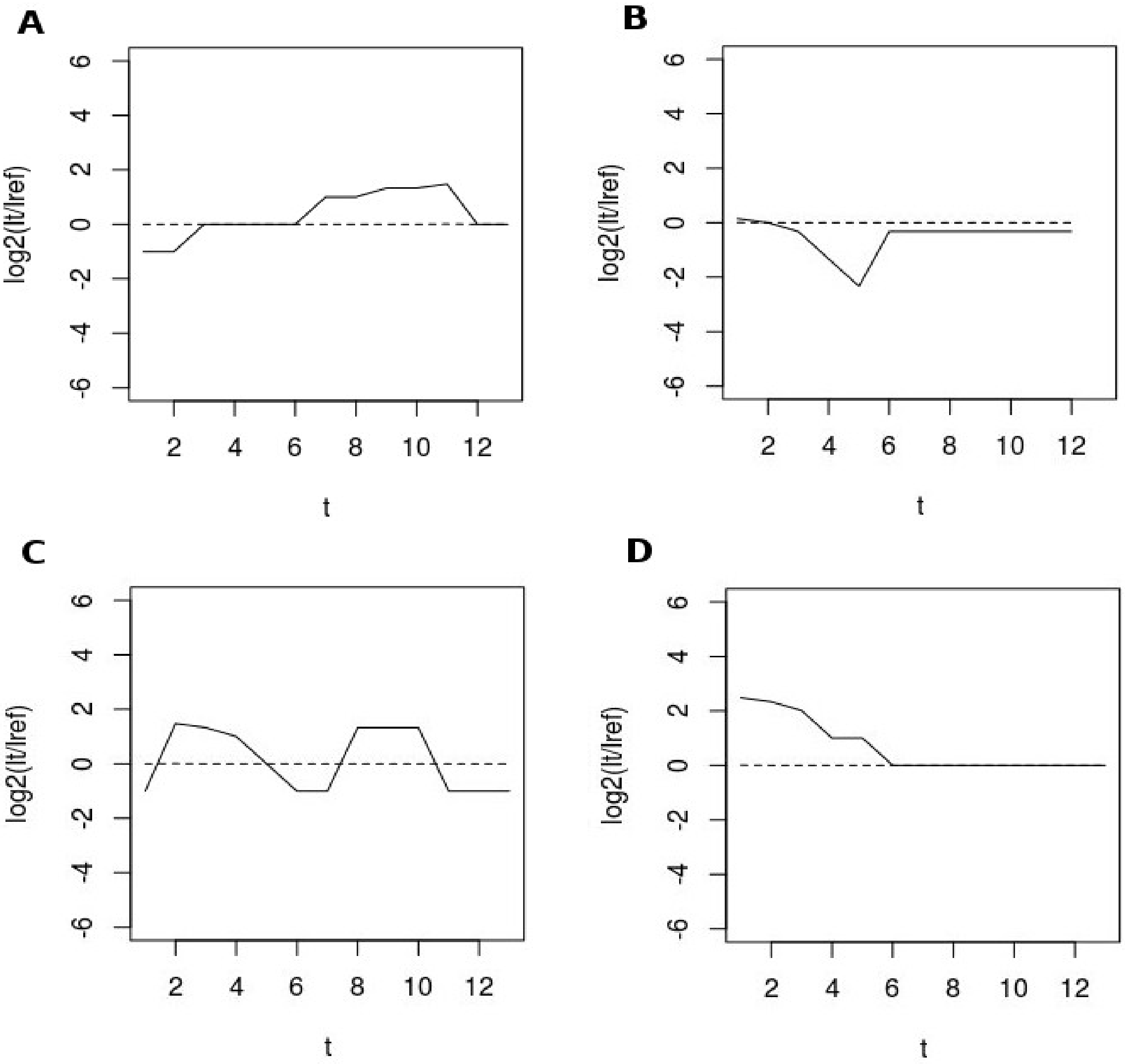

A
B
C
D
log2(It/Iref)
t
6
4
2
0
-2
-4
-6
2
4
6
8
10
12

E

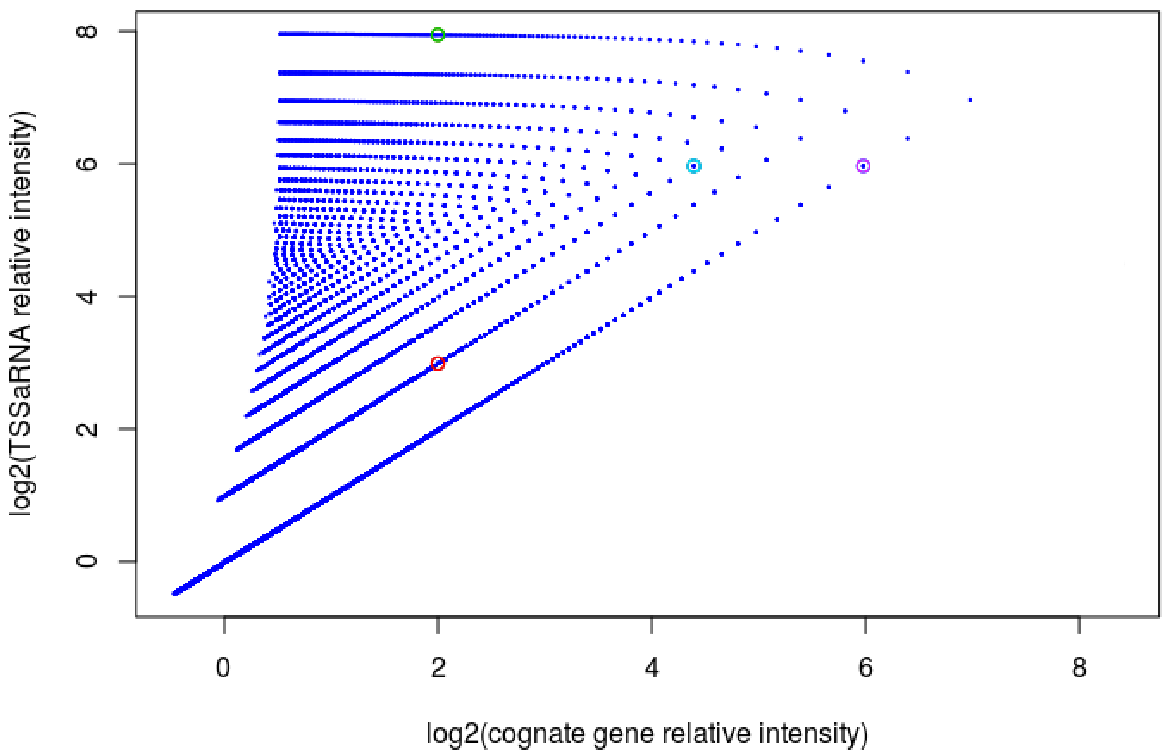

Figure S8 – Simple RNA polymerase pausing computational model simulation. Simulated expression profiles of TSSaRNAs differentially expressed during a growth curve relative to the control condition. Tiling microarray output simulation for a 35bp TSSaRNA in a 2kb cognate gene. Varying the only two model parameters it is possible to generate situations in which the cognate gene expression level remains constant over time and TSSaRNA levels can vary almost arbitrarily. Panels A to D are build mimicking our experimental setup displayed in manuscript's Figures 4 and Figure S7 exploring the parameter space. Vertical-axis – log2 ratios between simulated quantity of transcripts in each time point (I_t) and the amount simulated at reference time-point (I_{ref}). Black solid line – TSSaRNA expression profile. Black dashed line – cognate gene expression profile (constant over time and arbitrarily set to the same value of reference condition). Panel E shows all kinds of log2 rations that can be obtained for a TSSaRNA probe and its cognate gene when scanning the parameter space: $\Delta\tau$ and Δt, "intrinsic transcription initiation interval" and "time spent stalled", respectively. This example scans $\Delta\tau$ from 2 to 700 time units, Δt from 3 to 700 time units and simulates a 2kb gene with a 35bp TSSaRNA associated. Highlighted points in Panel E are examples of relatively constant TSSaRNA levels with an appropriately 3-fold difference in cognate gene level (light blue and purple circles, corresponding to Δt = 46 and $\Delta\tau$ = 12 time units and Δt = 14 and $\Delta\tau$ = 8 time units, respectively), and a 32-fold difference in TSSaRNA levels with relatively constant cognate gene levels (red and green circles, corresponding to Δt = 250 and $\Delta\tau$ = 4 time units and Δt = 250 and $\Delta\tau$ = 84 time units, respectively). Qualitatively, almost any complex dynamical behavior can be obtained if the pausing rhythm and the RNA polymerase arriving rate are jointly regulated by environmental clues.
