# Supplementary material for: Transcription Start Site Associated RNAs (TSSaRNAs) Are Ubiquitous in All Domains of Life
Source: PLoS One. 2014 Sep 19;9(9):e107680. doi: 10.1371/journal.pone.0107680 (PMC4169567; doi:10.1371/journal.pone.0107680)
Supplement: Figure S9 — Illustration of the TSSaRNA identification procedure. The yellow arrow represents a cognate gene's coding sequence region (CDS). Green vertical bars represent mapped reads' start coordinates and their abundances. The grey circle highlights the most frequent start coordinate. The grey box zoom illustrates the set of reads which mapped to this specific coordinate position, composed by two populations: identical reads from TSSaRNAs and other reads that originate from cognate gene transcripts. Black horizontal bars represent regions, relative to translation initiation site position (start codon position), around which the search for the most frequent start coordinate position was performed. (PDF) [file pone.0107680.s009.pdf]

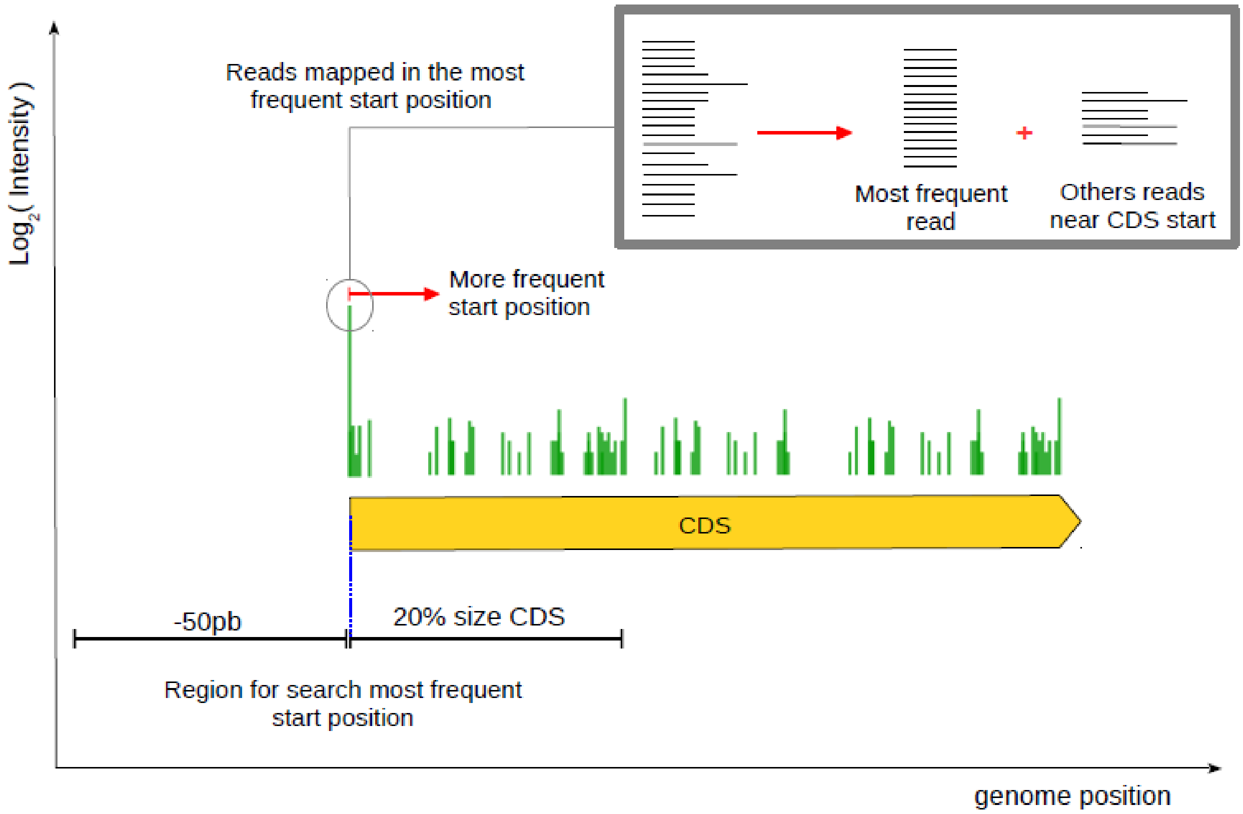

Figure S9 – Illustration of the TSSaRNA identification procedure. The yellow arrow represents a cognate gene's coding sequence region (CDS). Green vertical bars represent mapped reads' start coordinates and their abundances. The grey circle highlights the most frequent start coordinate. The grey box zoom illustrates the set of reads which mapped to this specific coordinate position, composed by two populations: identical reads from TSSaRNAs and other reads that originate from cognate gene transcripts. Black horizontal bars represent regions, relative to translation initiation site position (start codon position), around which the search for the most frequent start coordinate position was performed.
