# Supplementary material for: Transcription Start Site Associated RNAs (TSSaRNAs) Are Ubiquitous in All Domains of Life
Source: PLoS One. 2014 Sep 19;9(9):e107680. doi: 10.1371/journal.pone.0107680 (PMC4169567; doi:10.1371/journal.pone.0107680)
Supplement: Figure S10 — Illustration of the probe selection for TSSaRNA differential expression analysis. Light blue horizontal bars illustrate tiling array probe intensities for the reference condition. The best probe that represent the TSSaRNA is highlighted in red. The yellow arrow represents a cognate gene. Black dashed lines represent the TSSaRNA boundaries defined by RNA-seq. Gray boxes represent the regions used to calculate the neighbourhood expression intensity, which was compared to the TSSaRNA probe. (PDF) [file pone.0107680.s010.pdf]

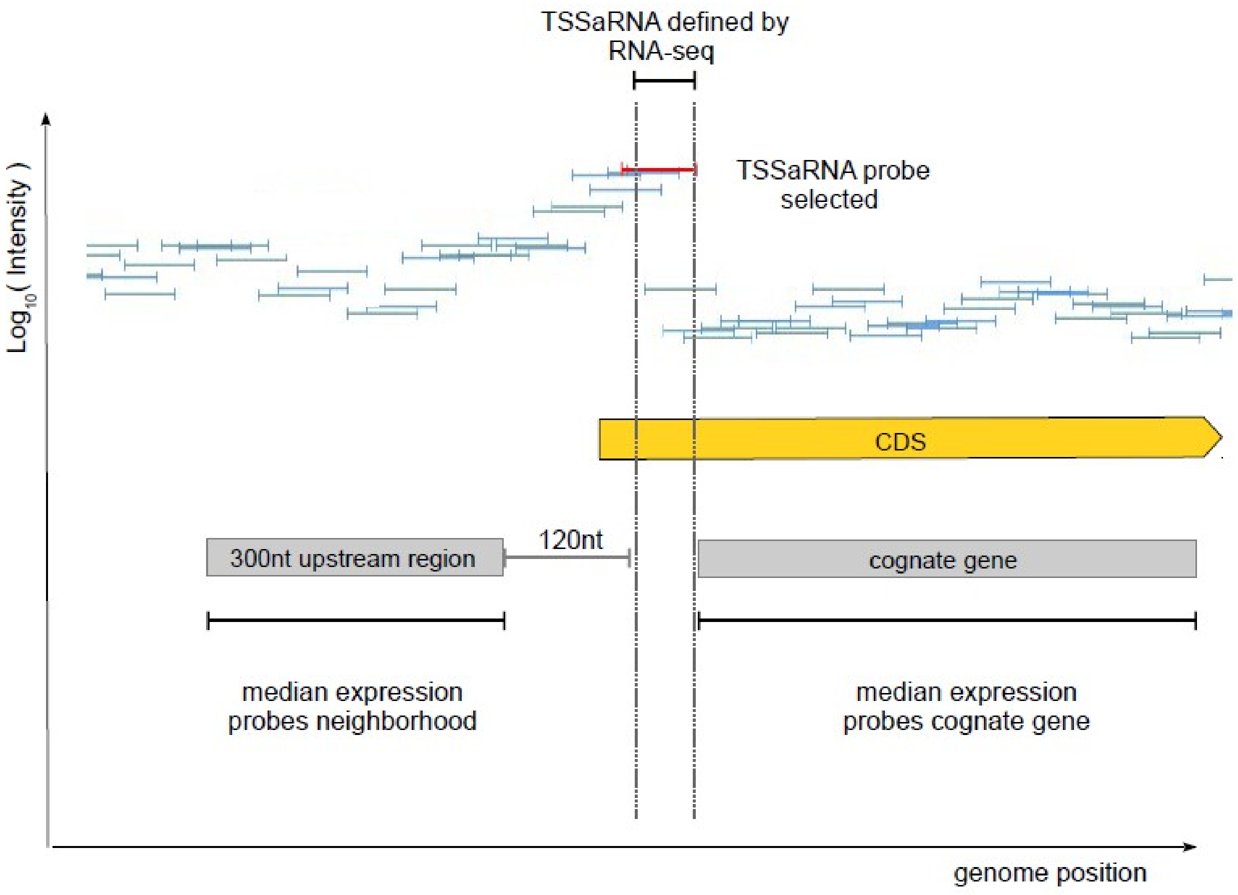


**Figure S10 – Illustration of the probe selection for TSSaRNA differential expression analysis.** Light blue horizontal bars illustrate tiling array probe intensities for the reference condition. The best probe that represent the TSSaRNA is highlighted in red. The yellow arrow represents a cognate gene. Black dashed lines represent the TSSaRNA boundaries defined by RNA-seq. Gray boxes represent the regions used to calculate the neighbourhood expression intensity, which was compared to the TSSaRNA probe.
