# Supplementary material for: Transcription Start Site Associated RNAs (TSSaRNAs) Are Ubiquitous in All Domains of Life
Source: PLoS One. 2014 Sep 19;9(9):e107680. doi: 10.1371/journal.pone.0107680 (PMC4169567; doi:10.1371/journal.pone.0107680)
Supplement: Figure S11 — Illustration of the method used to define a differentially expressed TSSaRNA in H. salinarum. The three-dimensional tiling microarray data (relative intensities vs growth-curve vs genome loci) is reduced to two dimensions and then to a single representative value. A TSSaRNA expression profile is considered distinct from its cognate gene if there are at least two time-points in which their relative intensities are at least 10-fold apart. Relative intensities are considered between a time-point and the reference growth condition. Relative intensities for TSSaRNAs are provided by the best tiling array probe (red horizontal bar) to fit a RNA-seq based TSSaRNA boundaries definition (see Figure S10). Relative intensities for their cognate genes are provided by the median of all non-overlaping adjacent tiling array probes (horizontal magenta dashed line). From all time-points along the growth-curve, the final differential expression value to be reported is from the data slice (fold-change vs position projection) where the 2nd top difference between TSSaRNA and cognate gene is found (blue vertical dashed line at t = 5). (PDF) [file pone.0107680.s011.pdf]

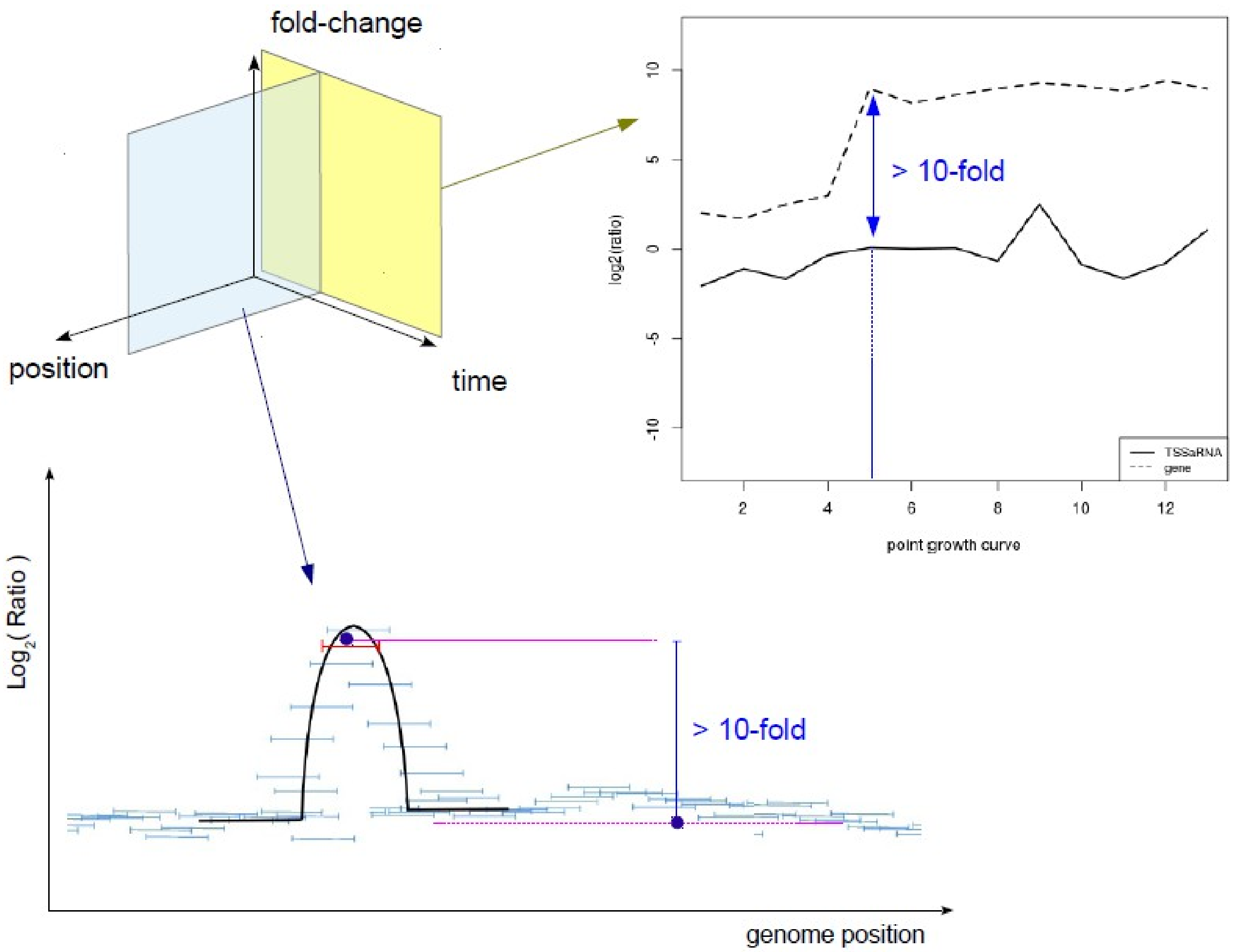

Figure S11 – Illustration of the method used to define a differentially expressed TSSaRNA in *H. salinarum*. The three-dimensional tiling microarray data (relative intensities vs growth-curve vs genome loci) is reduced to two dimensions and then to a single representative value. A TSSaRNA expression profile is considered distinct from its cognate gene if there are at least two time-points in which their relative intensities are at least 10-fold apart. Relative intensities are considered between a time-point and the reference growth condition. Relative intensities for TSSaRNAs are provided by the best tiling array probe (red horizontal bar) to fit a RNA-seq based TSSaRNA boundaries definition (see Figure S10). Relative intensities for their cognate genes are provided by the median of all non-overlaping adjacent tiling array probes (horizontal magenta dashed line). From all time-points along the growth-curve, the final differential expression value to be reported is from the data slice (fold-change vs position projection) where the 2nd top difference between TSSaRNA and cognate gene is found (blue vertical dashed line at t=5).
